# Effectiveness of Hydrotherapy on Neuropathic Pain and Pain Catastrophization in Patients With Spinal Cord Injury: Protocol for a Pilot Trial Study

**DOI:** 10.2196/37255

**Published:** 2022-04-29

**Authors:** Andrés Reyes Campo, Sara Gabriela Pacichana-Quinayáz, Francisco Javier Bonilla-Escobar, Luz Miriam Leiva-Pemberthy, Maria Ana Tovar-Sánchez, Olga Marina Hernández-Orobio, Gloria-Patricia Arango-Hoyos, Adnan Mujanovic

**Affiliations:** 1 Departamento de Medicina Física y Rehabilitación Grupo de Investigación en Rehabilitación de la Universidad del Valle, Universidad del Valle Hospital Universitario del Valle Cali Colombia; 2 Fundación Somos Ciencia al Servicio de la Comunidad, Fundación SCISCO/Science to Serve the Community Foundation, SCISCO Foundation Cali Colombia; 3 Institute for Clinical Research Education School of Medicine University of Pittsburgh Pittsburgh, PA United States; 4 Grupo de Investigación SINERGIA Escuela de Rehabilitación Humana Universidad del Valle Cali Colombia; 5 Department of Diagnostic and Interventional Neuroradiology University Hospital Bern Inselspital Bern Switzerland

**Keywords:** spinal cord injury, neuropathic pain, quality of life, catastrophization, hydrotherapy, neurology, spinal cord, nonpharmacological

## Abstract

**Background:**

Neuropathic pain (NP) is one of the most frequent spinal cord injury (SCI) complications. Pain, quality of life, and functionality are associated and can lead to pain catastrophization. Pharmacological management of patients with NP secondary to SCI is widely known and there is increasing evidence in the area. Nevertheless, nonpharmacological management is not fully elucidated since its efficacy is inconclusive.

**Objective:**

We hypothesize that (1) hydrotherapy is effective in reducing NP secondary to SCI. Additionally, our secondary hypotheses are that (2) hydrotherapy decreases the catastrophization of NP, and that (3) hydrotherapy improves life quality and minimizes the degree of disability, when compared to physical therapy.

**Methods:**

A sample of approximately 20 participants will be randomly assigned to either the intervention (hydrotherapy) or control group (standard physical therapy). Both interventions will be administered twice a week over a 9-week period (18 sessions in total). Primary outcomes are changes in neuropathic pain perception and pain catastrophization. Secondary outcomes are changes in disability and quality of life scores. They will be assessed at baseline and follow-up at 4 weeks after discharge. Validated Spanish language scales that will be used are the following: Numerical Pain Rating Scale, Pain Catastrophization, Health-related Quality of life, and the World Health Organization’s Disability Assessment Schedule 2.0. Generalized mixed linear models will be used for comparing baseline and postintervention means of each group and their differences, together with 95% CIs and *P* values. A *P* value of less than .05 will be considered significant.

**Results:**

Recruitment began in April 2019, and we recruited the last participants by December 2019, with 10 individuals assigned to hydrotherapy and 8 to physical therapy (control). Results from this study will be disseminated via scientific publication, in ClinicalTrials.gov, and in national and international conferences in the latter half of 2022.

**Conclusions:**

This trial will explore the effects of hydrotherapy on neuropathic pain, together with functionality and quality of life, in patients with SCI. Furthermore, this study aims to evaluate these therapeutic modalities, including perception variables, and mental processes, which may affect the clinical condition and rehabilitation outcomes in these patients. Hydrotherapy is likely to be a safe, efficient, and cost-effective alternative to the current standard of care for NP secondary to SCI, with comparable results between the two.

**Trial Registration:**

ClinicalTrials.gov NCT04164810; https://clinicaltrials.gov/ct2/show/NCT04164810

**International Registered Report Identifier (IRRID):**

DERR1-10.2196/37255

## Introduction

### Background

Neuropathic pain (NP) is one of the most frequent spinal cord injury (SCI) complications, with the most negative impact on quality of life [1]. Patients with NP show significant differences in quality of life when compared to those without it [2]. The prevalence of NP secondary to SCI varies depending on the population characteristics, chronicity of the lesion, and the criteria of defining it. In a meta-analysis, the prevalence of NP in patients SCI was 53% (95% CI 38.58-67.47) [3], with a prevalence of 47.8% being observed in Cali, Colombia [4-6]. Pain, quality of life, and functionality are proven to be associated, and patients with secondary NP to SCI are affected in these areas [7,8], thus limiting participation in daily activities and self-care. Chronic pain has an effect on emotional state, as well as cognition, in regard to pain anticipation, which can further lead to catastrophization.

The term “pain catastrophization” is of a recent use, consisting of three components: rumination (putting excessive focus on the painful sensation), magnification (exaggerating the damage), and perception of inability to control the symptom [9]. Different strategies have been proposed to alleviate the complications of SCI [10-12]. The pharmacological management of patients with NP secondary to SCI is widely known, and there is increasing evidence of anticonvulsant and antidepressant use for its management [13,14]. Nevertheless, the nonpharmacological management is not fully elucidated since the efficacy is inconclusive. Previous studies reported interventions such as transcranial electrical stimulation, acupuncture, and transcutaneous electrical nerve stimulation [15,16], which improved NP in patients with SCI; however, more evidence on other nonpharmacological interventions is needed.

The physiological mechanisms of hydrotherapy on pain are well established [17,18], and there is evidence of its use in the management of painful syndromes such as fibromyalgia and chronic lower back pain [19-21]. In patients with multiple sclerosis, it has been found that hydrotherapy improves quality of life [22]; this also holds true for patients with mild Parkinson disease, where hydrotherapy had improvements on functional mobility [23,24]. Additionally, there have been registered improvements in active neck mobility in cases of cervical dystonia [25] and in postural balance of patients with stroke [26,27]. The effects of hydrotherapy in patients with NP secondary to SCI are currently unknown.

### Primary and Secondary Objectives

The main study hypothesis is that (1) hydrotherapy is effective in reducing NP in patients with SCI. Additionally, our secondary hypotheses are that (2) hydrotherapy decreases the catastrophization of neuropathic pain, and that (3) hydrotherapy improves life quality and minimizes the degree of disability, when compared to the other physical therapy techniques.

## Methods

### Trial Design

We will carry out a single-masked pilot trial of parallel groups in usual clinical practice conditions, with an allocation ratio of 1:1 (SPIRIT checklist in Multimedia Appendix 1 and the World Health Organization’s Trial Registration Data Set in Multimedia Appendix 2).

### Participants

Participants will be recruited in two strategies: the first one involves screening eligible people in the SCI clinic in a prospective manner among those with appointments, and the second one involves calling participants in a follow-up call from the spinal cord service to check for their eligibility (Multimedia Appendix 3). Those eligible to participate in the study are patients who have a confirmed diagnosis of NP secondary to trauma in accordance with the International Spinal Cord Injury Pain classification [28], based on their clinical evaluation and physical examination, and those who meet the following criteria: ≥18 years of age, Douleur Neuropathique-4 (DN-4) score of ≥4 [29], and level of injury below C3. Exclusion criteria are as follows: active pressure ulcers, grade E of the American Spinal Cord Injury Association (ASIA) classification [28], cognitive impairment, ostomies, permanent bladder catheter, signs of systemic inflammatory response or urinary symptoms, major surgery in the past 2 months, noncontrolled hypertension (defined as a systolic blood pressure of >185 mm Hg or a diastolic blood pressure of >110 mm Hg refractory to treatment), active dyspeptic ulcer, severe liver fibrosis or portal hypertension, acute pericarditis or pancreatitis, known renal failure or requirement of hemodialysis or peritoneal dialysis, in-hospital stroke, and current participation in another clinical trial.

### Medical Assessment

The evaluation will begin with sociodemographic questions and presence of complications secondary to the SCI (participant questionnaire in Multimedia Appendix 4). In addition, the checklist of exclusion criteria for participation in the study will be verified (checklist of inclusion and exclusion criteria in Multimedia Appendix 5). In this initial evaluation, first measurement (baseline) will be performed. The medical examination will last 20 minutes and will be performed by a resident of the physical medicine and rehabilitation program. Once the medical evaluation is finished, the participants will be randomized into the control or intervention groups.

### Randomization

Participants will be assigned to one of the 2 groups by means of random blocks of 4, constructed with all possible combinations of the order of assignment based on random numbers (randomization protocol in Multimedia Appendix 6). The randomization list will remain with the coordinator for the whole duration of the study. Thus, randomization will be conducted without any influence of the principal investigators, interviewers, clinical evaluators, or therapists. The data analyst will be masked of participants’ allocation.

### Assignment of Interventions

The groups compared in this study are the intervention (hydrotherapy) and control (standard physical therapy) groups. This control group was chosen as the current standard of care for nonpharmacological management of NP in SCI is physical therapy [30-32].

Participants will be assessed as follows: at the beginning of the intervention (baseline; Time 1) and at the end of the intervention (4 weeks after discharge; Time 2). Table 1 describes the schedule with procedures and the assessments scales in the trial timeline.

**Table 1:** Schedule of enrollment and assessments.

Time point (T)		T1: baseline (week 0)	T2: follow-up (week 4)
**Enrollment**			
	Project information: written or oral communication	✓	
	Written informed consent	✓	
	Eligibility assessment	✓	
	Randomization	✓	
**Assessment**			
	Douleur Neuropathique-4 items	✓	
**Primary outcomes**			
	Pain catastrophization Scale	✓	✓
	Numerical Pain Rating Scale	✓	✓
**Secondary outcomes**			
	Quality Short-Form Health Survey 36	✓	✓
	World Health Organization Disability Assessment Schedule 2.0	✓	✓

### Interventions

Participants in both groups will receive treatments twice a week for 9 weeks, resulting in a total of 18 sessions. Each session will last 1 hour. A 9-week period of interventions is considered because antidepressant and anticonvulsant drugs, which act on the intensity of neuropathic pain, cause effects after 3 weeks of administration. Since the pathophysiological mechanisms of interventions are similar [33], we are expecting to see initial effects only after this period. Additionally, previous studies with hydrotherapy and physical therapy also provided interventions in a 4-8–week period [34,35]. The second assessment has been scheduled 4 weeks after discharge since that time frame has proven as an adequate threshold for evaluating short-term training program efficacy in patients with SPI [30]. Participants will be scheduled for the therapy sessions and will be followed-up by a research coordinator who will remind the participants about their appointments.

The department of physical medicine and rehabilitation has 3 physical therapists specialized in hydrotherapy under different modalities, and a pool equipped to perform this type of intervention. The most commonly used techniques in the institution are Bad Ragaz [36] for motor control and the Watsu technique [37] for relaxation and spasticity management. The standard physical therapy protocol and the hydrotherapy protocol were reviewed by a panel of therapists and researchers. These protocols include technical and clinical adjustments to standardize therapeutic objectives of the interventions (standard physical therapy protocol in Multimedia Appendix 7 and the hydrotherapy protocol in Multimedia Appendix 8). Participants in any group can receive alternative treatments including medications for pain and physical therapy. They will inform researchers through the surveys and measurements of the study, so that the effects of those extra interventions in the analysis can be accounted for. Interventions will be discontinued if the participant requests it or owing to the appearance of exclusion criteria or severe adverse events (adverse event report form in Multimedia Appendix 9).

### Follow-up

After assignment to the intervention or control group, it is possible that participants voluntarily leave the study, withdraw owing to medical indications, are lost during follow-up, or deviate from the protocol. These will be defined in accordance with the CONSORT statement and will be recorded in a specific format for follow-up (form: causes of withdrawal in Multimedia Appendix 10) [38]. Participants who withdraw from the interventions will be invited to answer the follow-up questionnaire at least three times via phone call.

### Data Collection

To identify the presence of neuropathic pain, the DN-4 will be used [29], which consists of ten items: 7 related to pain characteristics through an interview and the other 3 items through a clinical examination. This questionnaire, which is validated in Spanish [39], consists of descriptions and pain signs, which are evaluated dichotomously (Yes/No) in order to identify patients who have a high probability of having NP. The scores of the individual items are added together to obtain a maximum total score of 10, with a cut-off point of ≥4 (participant questionnaire in Multimedia Appendix 4).

Primary outcomes will be assessed with the Pain Catastrophization Scale (PCS) [40], the Numerical Pain Rating Scale (NPRS) [41,42], and the ASIA impairment scale [43]. The PCS is an instrument validated in Spanish for pathologies such as fibromyalgia, amputees, and patients with NP following SCI [44,45]. It inquiries about the thoughts and feelings that arise in the presence of physical pain caused by diseases, wounds, surgeries, etc. This scale has 13 items rated on a Likert scale from 0 to 4 (0=nothing at all, 1=a little, 2=moderately, 3=a lot, and 4=all time) [40,46,47]. The NPRS is a scale used in various clinical settings for multiple health conditions and measures pain intensity subjectively through a rating of 0 to 10, where 0 indicates no pain and 10 indicates the worst pain ever experienced [41,42,48]. The ASIA impairment scale is used as a universal classification tool for SCI, determining the level of sensory and motor impairment on each side of the body, single neurological level of injury, and whether the injury is complete. It will be used to quantitate sensory testing, limited by the point upon which the level and nature of SCI are present [43].

Secondary outcomes will be assessed with the Spanish version of the Quality Short-Form Health Survey, a generic scale evaluated in numerous studies [49]. This scale assesses health-related quality of life, and has been used in patients with SCIs, among other health conditions [2,50,51]. It has 36 items distributed in subscales of physical functioning, physical role, body pain, general health, vitality, social function, emotional role, and mental health. It contains Likert-type and dichotomous (Yes/No) questions with a minimum score of 0 and maximum of 100. Similarly, disability will be assessed using the World Health Organization’s Disability Assessment Schedule 2.0 questionnaire, which inquiries about difficulties that the individual has owing to a particular health condition [52]. For this study, the 12-item version will be used, which is composed up of a Likert scale ranging from 1=“no difficulty” to 5=“extreme difficulty” or “cannot do it.” The scores will be averaged for analysis [52]. In addition, sociodemographic and clinical questions will be asked for the adjustment of the analysis in a general information format (participant questionnaire in Multimedia Appendix 4). There will not be a run-in or washout period.

### Data Management, Monitoring, and Auditing

The Steering Committee (SC) is formed by the team of researchers and presided by the principal investigator. It will meet on weekly basis to analyze the therapists’ clinical notes, which will be recorded after each session of therapy from both groups in order to verify participants’ attendance, their adaptation to therapy, adverse effects, contraindications, and clinical evolution. The follow-up survey (exit survey) will be scheduled 4 weeks after the end of the therapies in both groups. Participants will be assessed twice: in a baseline survey, and in a follow-up survey at the end of the interventions. To identify the survey as baseline or follow-up, the numbers “_0” or “_1” will be used at the end of the code of each survey to indicate the type of measurement. This database will be managed in accordance with the rules of security and confidentiality of the information, which govern the health care institution and Universidad del Valle. Any serious adverse event will be assessed and classified by the Data Safety Monitoring Committee. The trial will be ended using a *P* value of <.001 as a stop boundary. There will not be any interim analysis.

### Ethics Approval

The study is endorsed by the Department of Physical Medicine and Rehabilitation, Hospital Universitario del Valle, Cali, Colombia. It was approved by the institutional review board of Universidad del Valle and Hospital Universitario del Valle (Internal Code 143-018/research ethics approval act 011-018). Additionally, the research complies with ethical standards of the Declaration of Helsinki; and in Colombia, it complies with the Ministry of Health resolution number 8430 of 1993. Since this study is considered to have “greater than minimum risk,” as it is a pilot trial where clinical interventions are conducted, there is, among other things, a risk of falls when entering and leaving the pool or a presence of fungal skin infections due to moisture. Before starting the medical evaluation, written informed consent for the study will be presented to participants by trained interviewers. These interviewers are not part of the medical assessment or randomization. Participants who agree to participate will sign the consent form, together with the required signatures of two witnesses (informed consent in Multimedia Appendix 11). Patient identities will be kept confidential, and an alphanumeric code will be used to identify each patient without enabling the distribution of personal data. The information that will be obtained in this study will be kept confidential, its use will be exclusive to the researchers, and disaggregated data will not be presented or used in a particular way that may lead to disclosure of confidential information, violation of any of the participants’ rights, or to the participants feeling stigmatized or discriminated against.

### Availability of Data and Material

Data cannot be made publicly available since that would compromise confidentiality and might reveal the identity or location of participants. Additionally, public availability of data would be in violation of the Colombian Law 1581 of 2012 and the Regulatory Act 1377 of 2013 and Article 15 of the Colombian Constitution for treatment and protection of personal data. The SC could consider those requests once the data are fully collected, and identifiers have been destroyed with approval of the institutional review board of the participating institutions. There are no contractual agreements that limit investigators’ access to data.

### Statistical Analysis

#### Sample Size and Power Calculation

In order to demonstrate recruitment feasibility, 20 participants will be enrolled (10 per group) in accordance with sample size recommendations for pilot studies [53]. As this is a pilot study, power calculations were not performed.

#### Descriptive Analysis

The categorical variables will be described in terms of frequency and percentage, and for hypothesis testing, chi-square test or the Fisher exact test will be used. Quantitative variables will be described with measures of central tendency and dispersion, and hypothesis tests based on the type of variables to be analyzed as well as the assumptions of each test.

#### Effectiveness of the Intervention

Treatment effects will be derived using longitudinal modeling of within-person change in mean scores of the scales and subscales’ scores from baseline to follow-up. Baseline characteristics of participants in both groups will be compared using the chi-square or 2-tailed Fisher exact test for categorical variables, and the Student *t* test or Wilcoxon test for continuous variables, after assessing the tests’ assumptions. Statistical significance will be set at a 2-tailed α of .05, expressed as a 95% CI.

#### Analysis of Outcomes

An intent-to-treat analysis, which will include all study participants based on participants’ arm allocation will be used. A random effects model will be used to estimate the effect of hydrotherapy by including time points (0 = baseline, 1 = follow-up) and participant ID as random effects to account for within-person correlation across time and between-person correlation. Participants lost to follow-up will be handled using single imputation procedures on the basis of the amount of missing and their distribution.

## Results

**Figure 1:**
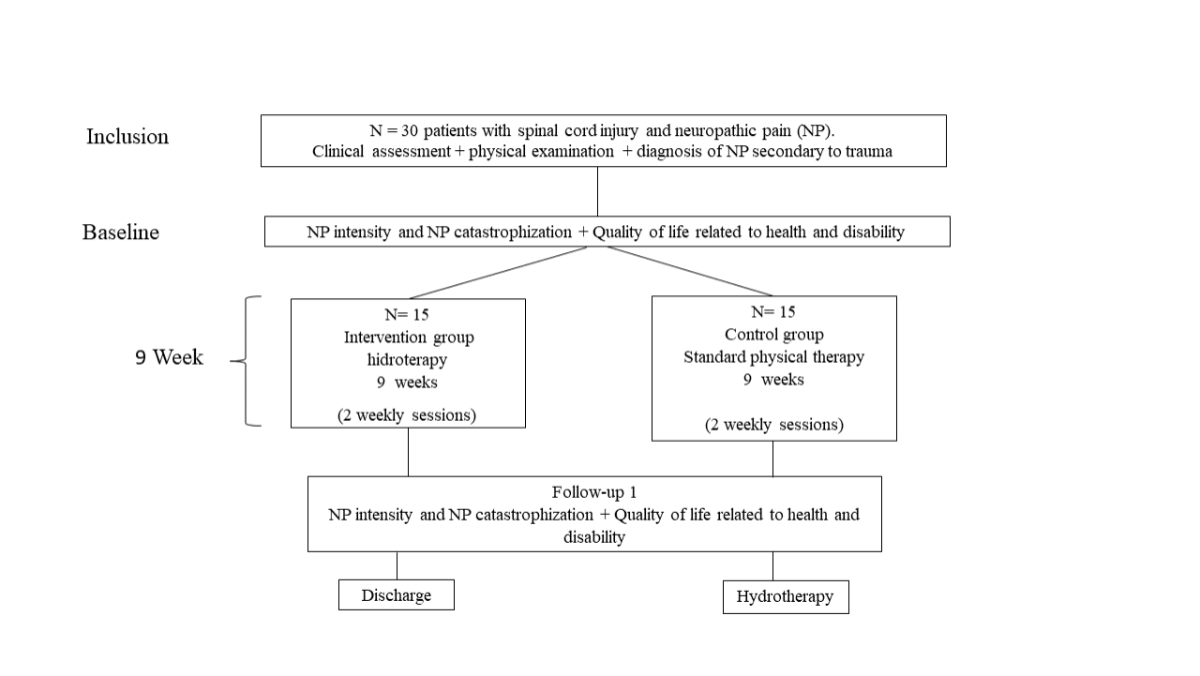
Flow of the study.

The first patient was enrolled in the study on March 30, 2019. The project timeline was changed owing to the COVID-19 pandemic and therefore delayed. Recruitment began in April 2019 and we recruited the final participants by December 2019. Figure 1 shows the flow diagram of the study. Participants were selected from the database of patients registered at the Spinal Trauma Clinic, Physical Medicine and Rehabilitation Unit of a Specialized Trauma Center [54]. We have collected information from 18 participants, including 10 in the hydrotherapy group and 8 in the physical therapy group. Results from the study will be disseminated via scientific publication, in ClinicalTrials.gov, and in national and international conferences in the second part of 2022. Authorship will be defined on the basis of the International Committee of Medical Journal Editors’ criteria for authorship. The authors do not have publication restrictions and will not use professional writers when reporting the results.

## Discussion

### Expected Findings

The expected main findings of this study are as follows: (1) effects on minimizing disability and improving quality of life between current standard and novel hydrotherapeutic approach are comparable; (2) hydrotherapy is a safe, efficient, and effective alternative to the current management of NP secondary to SCI; (3) given a choice, patients are more likely to choose hydrotherapy rather than the current standard of care if both options are presented to them. To our best knowledge, this is the first trial that proposes to evaluate the effects of hydrotherapy in the NP reduction in Latin America.

The purpose of rehabilitation is to maximize patients’ daily functional independence through a goal-oriented treatment [55]. Heterogenous results from previous hydrotherapeutic studies on patients with diabetic or other metabolic neuropathies do not allow for definitive conclusions [56]. Conversely, the hydrotherapeutic approach for patients with peripheral neuropathies has yielded positive results on gait and balance, which, besides pain, are the most commonly presented symptoms in our patient cohort [6,57].

The presence of a control group is necessary, since there are no currently available nonpharmacological treatments with evidence for NP management in patients with SCI. The pilot trial design with parallel groups aims to provide preliminary evidence on the clinical efficacy of hydrotherapy on NP secondary to SCI, with a single-masked study design being performed in order to alleviate bias. Consideration of a DN-4 score of ≥4 out of 10 for the identification of NP and mixed pain syndromes [39] ensures adequate screening in our trial population. This questionnaire has been suggested for epidemiological studies, where accurate identification of NP symptoms is also crucial to test in order improve therapeutic approaches.

Assessment of an alternative treatment to pharmacological management of neuropathic pain is reasonable, considering preclinical evidence on neuroendocrine changes facilitated by aquatic treatment, at the level of encephalin, corticotropins, endorphins, and prolactin, who are responsible for conduction of pain stimuli [58]. Accordingly, it may improve the prognosis of pain and its catastrophization [9,59]. Furthermore, this study will explore the evaluation of these therapeutic modalities, including variables related to perception and mental processes, which may affect the clinical condition and rehabilitation outcomes of these patients [46].

### Limitations

This study has some limitations. Given the single-center design of our study, the generalizability of our results will be limited. Even though we have accounted for patients lost to follow-up, true percentages might be higher than expected, therefore skewing our findings. Despite the advantages of a single-masked trial design, double-masked trials are prone to less pitfalls [60].

### Conclusions

Hydrotherapy is likely to be a safe, efficient, and cost-effective alternative to the current standard of care for NP secondary to SCI, with comparable results between the two.

Since this is a pilot trial, randomized controlled trials with calculated power levels and sample sizes will be warranted in order to provide conclusive answers on noninferiority or superiority of the proposed treatment effect.

## Appendix

Multimedia Appendix 1 SPIRIT checklist.

Multimedia Appendix 2 World Health Organization trial registration data set.

Multimedia Appendix 3 Telephone contact protocol.

Multimedia Appendix 4 Participant questionnaire.

Multimedia Appendix 5 Checklist of inclusion and exclusion criteria.

Multimedia Appendix 6 Randomization protocol.

Multimedia Appendix 7 Standard physical therapy protocol.

Multimedia Appendix 8 Hydrotherapy protocol.

Multimedia Appendix 9 Adverse event report form.

Multimedia Appendix 10 Form: cause for withdrawal.

Multimedia Appendix 11 Informed consent.

Multimedia Appendix 12 Peer review report 1 from the Universidad del Valle - Vicerrectoría de Investigaciones (Cali, Colombia).

Multimedia Appendix 13 Peer review report 2 from the Universidad del Valle - Vicerrectoría de Investigaciones (Cali, Colombia).

## Abbreviations

ASIA: American Spinal Cord Injury Association
DN-4: Douleur Neuropathique-4
NP: neuropathic pain
NPRS: Numerical Pain Rating Scale
PCS: Pain Catastrophizing Scale
SC: Steering Committee
SCI: spinal cord injury

## References

[1] Adriaansen J, Ruijs L, van Koppenhagen CF, van Asbeck FWA, Snoek G, van Kuppevelt D, Visser-Meily J, Post M (2016). Secondary health conditions and quality of life in persons living with spinal cord injury for at least ten years. J Rehabil Med.

[2] Westgren N, Levi R (1998). Quality of life and traumatic spinal cord injury. Arch Phys Med Rehabil.

[3] Burke D, Fullen B, Stokes D, Lennon O (2017). Neuropathic pain prevalence following spinal cord injury: A systematic review and meta-analysis. Eur J Pain.

[4] Reyes Campo W, Pacichana Quinayáz S, Bonilla Escobar F, Tovar Sánchez M (2018). Neuropathic pain in spinal cord injury population in Cali, Colombia. Ann Phys Rehabil Med.

[5] Giraldo YA, Castro JL, Tovar-Sánchez MA, Kumar AA, Pacichana-Quinayáz SG, Bonilla-Escobar FJ (2021). Epidemiology of traumatic spinal cord injuries in Colombia. Spinal Cord Ser Cases.

[6] Reyes-Campo A, Pacichana-Quinayás SG, Kumar AA, Leiva-Pemberthy LM, Tovar-Sánchez MA, Bonilla-Escobar FJ (2022). Factors associated with neuropathic pain in Colombian patients with spinal cord injury of traumatic origin: case-control study. Spinal Cord Ser Cases.

[7] Alschuler KN, Jensen MP, Sullivan-Singh SJ, Borson S, Smith AE, Molton IR (2013). The association of age, pain, and fatigue with physical functioning and depressive symptoms in persons with spinal cord injury. J Spinal Cord Med.

[8] Cohen JT, Marino RJ, Sacco P, Terrin N (2012). Association between the functional independence measure following spinal cord injury and long-term outcomes. Spinal Cord.

[9] Wade J, Riddle D, Price D, Dumenci L (2011). Role of pain catastrophizing during pain processing in a cohort of patients with chronic and severe arthritic knee pain. Pain.

[10] Huang KT, Lu Y (2021). Traumatic Spinal Cord Disorders: Current Topics and Future Directions. Semin Neurol.

[11] Park S, Yozbatiran N (2021). A Medical Student’s Perspective on the Growing Importance of Telemedicine/Telerehabilitation. Int J Med Students.

[12] Bangura A, Shuler T, Wright L, Lake A (2021). Spinal Cord Injury Induced Osteoporosis: Case Report and Current Literature. Int J Med Students.

[13] Finnerup NB, Attal N, Haroutounian S, McNicol E, Baron R, Dworkin RH, Gilron I, Haanpää M, Hansson P, Jensen TS, Kamerman PR, Lund K, Moore A, Raja SN, Rice ASC, Rowbotham M, Sena E, Siddall P, Smith BH, Wallace M (2015). Pharmacotherapy for neuropathic pain in adults: a systematic review and meta-analysis. Lancet Neurol.

[14] Hagen EM, Rekand T (2015). Management of Neuropathic Pain Associated with Spinal Cord Injury. Pain Ther.

[15] Fattal C, Kong-A-Siou D, Gilbert C, Ventura M, Albert T (2009). What is the efficacy of physical therapeutics for treating neuropathic pain in spinal cord injury patients?. Ann Phys Rehabil Med.

[16] Nardone R, Höller Y, Leis S, Höller P, Thon N, Thomschewski A, Golaszewski S, Brigo F, Trinka E (2014). Invasive and non-invasive brain stimulation for treatment of neuropathic pain in patients with spinal cord injury: a review. J Spinal Cord Med.

[17] Bender T, Karagülle Z, Bálint GP, Gutenbrunner C, Bálint PV, Sukenik S (2005). Hydrotherapy, balneotherapy, and spa treatment in pain management. Rheumatol Int.

[18] Hama A, Sagen J (2009). Antinociceptive effects of the marine snail peptides conantokin-G and conotoxin MVIIA alone and in combination in rat models of pain. Neuropharmacology.

[19] Baena-Beato PÁ, Artero EG, Arroyo-Morales M, Robles-Fuentes A, Gatto-Cardia MC, Delgado-Fernández M (2014). Aquatic therapy improves pain, disability, quality of life, body composition and fitness in sedentary adults with chronic low back pain. A controlled clinical trial. Clin Rehabil.

[20] Naumann J, Sadaghiani C (2014). Therapeutic benefit of balneotherapy and hydrotherapy in the management of fibromyalgia syndrome: a qualitative systematic review and meta-analysis of randomized controlled trials. Arthritis Res Ther.

[21] Waller B, Lambeck J, Daly D (2009). Therapeutic aquatic exercise in the treatment of low back pain: a systematic review. Clin Rehabil.

[22] Kargarfard M, Etemadifar M, Baker P, Mehrabi M, Hayatbakhsh R (2012). Effect of aquatic exercise training on fatigue and health-related quality of life in patients with multiple sclerosis. Arch Phys Med Rehabil.

[23] Ayán C, Cancela J (2012). Feasibility of 2 different water-based exercise training programs in patients with Parkinson's disease: a pilot study. Arch Phys Med Rehabil.

[24] Marinho-Buzelli AR, Bonnyman AM, Verrier MC (2015). The effects of aquatic therapy on mobility of individuals with neurological diseases: a systematic review. Clin Rehabil.

[25] Useros-Olmo AI, Collado-Vázquez S (2010). [Effects of an hydrotherapy program in the treatment of cervical dystonia. A pilot study]. Rev Neurol.

[26] Tripp F, Krakow K (2014). Effects of an aquatic therapy approach (Halliwick-Therapy) on functional mobility in subacute stroke patients: a randomized controlled trial. Clin Rehabil.

[27] Zhu Z, Cui L, Yin M, Yu Y, Zhou X, Wang H, Yan H (2016). Hydrotherapy vs. conventional land-based exercise for improving walking and balance after stroke: a randomized controlled trial. Clin Rehabil.

[28] Bryce TN, Biering-Sørensen F, Finnerup NB, Cardenas DD, Defrin R, Lundeberg T, Norrbrink C, Richards JS, Siddall P, Stripling T, Treede R, Waxman SG, Widerström-Noga E, Yezierski RP, Dijkers M (2012). International spinal cord injury pain classification: part I. Background and description. March 6-7, 2009. Spinal Cord.

[29] Bouhassira D, Attal N, Alchaar H, Boureau F, Brochet B, Bruxelle J, Cunin G, Fermanian J, Ginies P, Grun-Overdyking A, Jafari-Schluep H, Lantéri-Minet M, Laurent B, Mick G, Serrie A, Valade D, Vicaut E (2005). Comparison of pain syndromes associated with nervous or somatic lesions and development of a new neuropathic pain diagnostic questionnaire (DN4). Pain.

[30] Devillard X, Rimaud D, Roche F, Calmels P (2007). Effects of training programs for spinal cord injury. Ann Readapt Med Phys.

[31] Felix ER (2014). Chronic neuropathic pain in SCI: evaluation and treatment. Phys Med Rehabil Clin N Am.

[32] Leitzelar BN, Koltyn KF (2021). Exercise and Neuropathic Pain: A General Overview of Preclinical and Clinical Research. Sports Med Open.

[33] Siddall PJ, Cousins MJ, Otte A, Griesing T, Chambers R, Murphy TK (2006). Pregabalin in central neuropathic pain associated with spinal cord injury: a placebo-controlled trial. Neurology.

[34] Kandel L Hydrotherapy Versus Physiotherapy for Short-term Rehabilitation After Primary TKR. ClinicalTrials.gov.

[35] Valenza M Hydrotherapy Intervention in Elderly With Knee Osteoarthritis. ClinicalTrials.gov.

[36] Ainslie T (2020). Hydrotherapy aquatic physiotherapy and the application of bad ragaz ring method. JAHC.

[37] Schitter AM, Fleckenstein J, Frei P, Taeymans J, Kurpiers N, Radlinger L (2020). Applications, indications, and effects of passive hydrotherapy WATSU (WaterShiatsu)-A systematic review and meta-analysis. PLoS One.

[38] Turner LS, Shamseer L, Altman DG, Weeks L, Peters J, Kober T, Dias S, Schulz KF, Plint AC, Moher D (2012). Consolidated standards of reporting trials (CONSORT) and the completeness of reporting of randomised controlled trials (RCTs) published in medical journals. Cochrane Database Syst Rev.

[39] Perez C, Galvez R, Huelbes S, Insausti J, Bouhassira D, Diaz S, Rejas J (2007). Validity and reliability of the Spanish version of the DN4 (Douleur Neuropathique 4 questions) questionnaire for differential diagnosis of pain syndromes associated to a neuropathic or somatic component. Health Qual Life Outcomes.

[40] Sullivan MJL, Bishop SR, Pivik J (1995). The Pain Catastrophizing Scale: Development and validation. Psychol Assess.

[41] Huskisson E (1974). MEASUREMENT OF PAIN. Lancet.

[42] Price DD, Bush FM, Long S, Harkins SW (1994). A comparison of pain measurement characteristics of mechanical visual analogue and simple numerical rating scales. Pain.

[43] Kirshblum SC, Burns SP, Biering-Sorensen F, Donovan W, Graves DE, Jha A, Johansen M, Jones L, Krassioukov A, Mulcahey M, Schmidt-Read M, Waring W (2011). International standards for neurological classification of spinal cord injury (revised 2011). J Spinal Cord Med.

[44] Quartana PJ, Campbell CM, Edwards RR (2009). Pain catastrophizing: a critical review. Expert Rev Neurother.

[45] Turner JA, Jensen MP, Warms CA, Cardenas DD (2002). Catastrophizing is associated with pain intensity, psychological distress, and pain-related disability among individuals with chronic pain after spinal cord injury. Pain.

[46] Campbell CM, Buenaver LF, Finan P, Bounds SC, Redding M, McCauley L, Robinson M, Edwards RR, Smith MT (2015). Sleep, Pain Catastrophizing, and Central Sensitization in Knee Osteoarthritis Patients With and Without Insomnia. Arthritis Care Res (Hoboken).

[47] Olmedilla Zafra A, Ortega Toro E, Abenza Cano L (2013). Validación de la escala de catastrofismo ante el dolor (Pain Catastrophizing Scale) en deportistas españoles. CPD.

[48] Bryce TN, Budh CN, Cardenas DD, Dijkers M, Felix ER, Finnerup NB, Kennedy P, Lundeberg T, Richards JS, Rintala DH, Siddall P, Widerstrom-Noga E (2007). Pain after spinal cord injury: an evidence-based review for clinical practice and research. Report of the National Institute on Disability and Rehabilitation Research Spinal Cord Injury Measures meeting. J Spinal Cord Med.

[49] Vilagut G, Ferrer M, Rajmil L, Rebollo P, Permanyer-Miralda G, Quintana JM, Santed R, Valderas JM, Ribera A, Domingo-Salvany A, Alonso J (2005). [The Spanish version of the Short Form 36 Health Survey: a decade of experience and new developments]. Gac Sanit.

[50] Nagoshi N, Kaneko S, Fujiyoshi K, Takemitsu M, Yagi M, Iizuka S, Miyake A, Hasegawa A, Machida M, Konomi T, Machida M, Asazuma T, Nakamura M (2016). Characteristics of neuropathic pain and its relationship with quality of life in 72 patients with spinal cord injury. Spinal Cord.

[51] Putzke JD, Richards J, DeVivo MJ (2001). Quality of life after spinal cord injury caused by gunshot. Arch Phys Med Rehabil.

[52] Üstün TB, Kostanjsek N, Chatterji S, Rehm J, World Health Organization (2010). Measuring Health and Disability: Manual for WHO Disability Assessment Schedule WHODAS 2.0.

[53] In J (2017). Introduction of a pilot study. Korean J Anesthesiol.

[54] Montoya Casella A, Peralta Pizza F, Montes Gonzales IT, Rivera Jd, Ordoñez Ja (2017). Heridas por arma de fuego penetrantes a columna vertebralxperiencia del Hospital Universitario del Valle, 2012-2014. Rev Gastrohnup.

[55] Carter GT (2005). Rehabilitation management of peripheral neuropathy. Semin Neurol.

[56] Streckmann F, Zopf EM, Lehmann HC, May K, Rizza J, Zimmer P, Gollhofer A, Bloch W, Baumann FT (2014). Exercise intervention studies in patients with peripheral neuropathy: a systematic review. Sports Med.

[57] Marinho-Buzelli AR, Bonnyman AM, Verrier MC (2015). The effects of aquatic therapy on mobility of individuals with neurological diseases: a systematic review. Clin Rehabil.

[58] Mooventhan A, Nivethitha L (2014). Scientific evidence-based effects of hydrotherapy on various systems of the body. N Am J Med Sci.

[59] Sansone RA, Watts DA, Wiederman MW (2014). Pain, pain catastrophizing, and history of intentional overdoses and attempted suicide. Pain Pract.

[60] Day SJ, Altman DG (2000). Statistics notes: blinding in clinical trials and other studies. BMJ.

