# Supplementary material for: Effectiveness of Hydrotherapy on Neuropathic Pain and Pain Catastrophization in Patients With Spinal Cord Injury: Protocol for a Pilot Trial Study
Source: JMIR Res Protoc. 2022 Apr 29;11(4):e37255. doi: 10.2196/37255 (PMC9107053; doi:10.2196/37255)
Supplement: Multimedia Appendix 2 [file resprot_v11i4e37255_app2.docx]

**Appendix 2. World Health Organization trial registration data set**

| **Data category** | **Information** |
| --- | --- |
| Primary registry and trial identifying number | ClinicalTrials.gov  NCT04164810 |
| Date of registration in primary registry | November 15, 2019 |
| Secondary identifying numbers | 143-018, 011-018 |
| Source(s) of monetary or material support | Universidad del Valle |
| Primary sponsor | Universidad del Valle |
| Secondary sponsor(s) | Fundación SCISCO/SCISCO Foundation |
| Contact for public queries | Sara Gabriela Pacichana-Quinayaz, FT, MSc.  SCISCO Foundation. [saragabriela.uv@gmail.com](mailto:saragabriela.uv@gmail.com) |
| Contact for scientific queries | Maria Ana Tovar-Sanchez, MD. Universidad del Valle. [ana.tovar@correounivalle.edu.co](mailto:ana.tovar@correounivalle.edu.co). Francisco Javier Bonilla-Escobar, MD, MSc. Universidad del Valle, University of Pittsburgh, [fjbonillaescobar@gmail.com](mailto:fjbonillaescobar@gmail.com) |
| Public title | Effectiveness of hydrotherapy on neuropathic pain and pain catastrophization in patients with spinal cord injury |
| Scientific title | Effectiveness of hydrotherapy on neuropathic pain and pain catastrophization in patients with spinal cord injury—pilot, single masked, controlled trial |
| Countries of recruitment | Colombia |
| Health condition(s) or problem(s) studied | Neuropathic pain in patients with spinal cord injury |
| Intervention(s) | Active comparator: hydrotherapy (18 sessions of 1 hour, 2 days per week for 9 weeks)  Control comparator: physical therapy (18 sessions of 1 hour, 2 days per week for 9 weeks) |
| Key inclusion and exclusion criteria | Ages eligible for study: ≥18 years  Sexes eligible for study: both  Accepts healthy volunteers: no  Inclusion criteria: Patient with spinal cord injury, Over 18 years of age, DN-score 4 (Douleur Neuropathique-4) equal to or greater than 4, level of injury below C3.  Exclusion criteria: Active pressure ulcers, E classification in the ASIA (American Spinal Cord Injury Association), Cognitive impairment, Ostomies, Permanent bladder catheter, Signs of systemic inflammatory response, Urinary symptoms |
| Study type | Interventional  Allocation: randomized  Intervention model: parallel assignment  Masking: single-masked (investigator, outcomes assessor)  Primary purpose: prevention  Phase III |
| Date of first enrolment | March 30, 2019 |
| Target sample size | 30 participants, 15 in each comparison group |
| Recruitment status | Recruitment finalized |
| Primary outcome(s) | Pain catastrophization Scale (PCS), Numerical Pain Rating Scale (NPRS) |
| Key secondary outcomes | Quality Short-Form Health Survey 36 (SF-36), WHODAS 2.0 |
