# Supplementary material for: Effectiveness of Hydrotherapy on Neuropathic Pain and Pain Catastrophization in Patients With Spinal Cord Injury: Protocol for a Pilot Trial Study
Source: JMIR Res Protoc. 2022 Apr 29;11(4):e37255. doi: 10.2196/37255 (PMC9107053; doi:10.2196/37255)
Supplement: Multimedia Appendix 3 [file resprot_v11i4e37255_app3.docx]

**Appendix 3. Telephone contact protocol**

STEP 1 PHONE CALL

Hello, am I speaking with___________? (Confirm identity with ID and date of birth)

You are talking to Dr. Andrés Reyes/Miriam Leiva, from the Hospital Universitario del Valle (Department of Physical Medicine and Rehabilitation). We are calling you because we are conducting a clinical follow-up to patients with spinal cord injury, to determine how their clinical evolution is going.

|  | Yes | No |
| --- | --- | --- |
| Do you want to participate in this clinical follow-up? |  |  |

***Interviewer****: If no, thank the participant. Ask for another opportunity to call. If yes, proceed with the interview*

Please tell me how is your health status at this moment? Excellent / Good / Poor / Bad / Very bad

Why? __________________________________________________________________

How are you feeling with your physical rehabilitation? Excellent / Good / Poor / Bad / Very bad

Why? __________________________________________________________________

When was the last time that you were seeing by our service (physical rehabilitation)? __________________________________________________________________

***Interviewer****: Address concerns from the patient, advice, invite the patient to schedule an appointment with the service.*

In addition to checking-in with you, we would like to invite you to a study that we are carrying out to test a therapy for pain caused by injuries of the spine.

Would you like to know more about the study: Yes / No

***Interviewer****: If the patient answer yes, proceed with the following questions. If no, thank the patient, inform that we are going to call again to follow-up with the rehabilitation as usual, and end the call.*

We are going to ask you a few questions about some symptoms that you may or may not have:

| Question | Yes | No |
| --- | --- | --- |
| 1. Did the spinal cord injury occurs more than 2 years ago? |  |  |
| 1. Are you 18 or more years old? |  |  |
| 1. Do you have any active ulcers pressure, ostomy? |  |  |
| 1. (identified by the interviewer) Impaired cognitive? |  |  |
| 1. Do you have pain with one or more of the following characteristics? |  |  |
| 1. Burning |  |  |
| 2. Painful cold |  |  |
| 3. Electric cramps |  |  |
| 1. Is this pain associated with one or more of the following symptoms in the same area? |  |  |
| 1. Tingling |  |  |
| 2. Pins and needles |  |  |
| 3. Numbness |  |  |
| 4. Itching |  |  |
| 1. Have you started or adjusted your pharmacological management dose for neuropathic pain in the last 4 weeks? |  |  |
| 1. Do you have a gastrostomy, tracheostomy, or permanent urinary catheter? |  |  |

**NOT eligible**

If the participant answers NO, in A **or** B

If the participant answers YES in C **or** D

If the participant answer YES in H

**Eligible**

If the participant answers YES, in A **and** B*, continue with the questions.*

If the participant answers YES to **at least** 2 of the 7 characteristics (E and F), they *are eligible to participate in the study.*

Question G is for information only.

***Interviewer****: Thank the participant and inform them about the eligibility.*

Thank you so much (patients name), then we will schedule a medical appointment for the physical evaluation.

(Offered schedule options - date and time – for physical evaluation appointment).

IF THE PARTICIPANT **WAS NOT ELIGIBLE**

Thank you very much, we appreciate your time.

STEP 2 SURVEY PATIENTS

Once the physical examination is complete, the main questionnaire will be applied, which included the trial instruments.

STEP 3 ENTER THE DATA

Once the questionnaire quality data is verified, the information will be entered into the project database.
