# Supplementary material for: Effectiveness of Hydrotherapy on Neuropathic Pain and Pain Catastrophization in Patients With Spinal Cord Injury: Protocol for a Pilot Trial Study
Source: JMIR Res Protoc. 2022 Apr 29;11(4):e37255. doi: 10.2196/37255 (PMC9107053; doi:10.2196/37255)
Supplement: Multimedia Appendix 4 [file resprot_v11i4e37255_app4.docx]

**Appendix 4. Participant questionnaire**

1. **SECTION A: PARTICIPANT IDENTIFICATION**

| Day | | Month | | Year | | | |
| --- | --- | --- | --- | --- | --- | --- | --- |
|  |  |  |  |  |  |  |  |

Date:

**Survey Check Box**

Comments:

Participants´s ID (Request the coordinator): __________

Interviewer´s ID (Request the coordinator): ___________

Measure (*Check mark √,* only one)

| 1. First Measure (ALL+DN-4) _______ 2. Second Measure (ALL) _______ |
| --- |

Start Time: ____________________

Phone/Cell1:______________________ Phone/Cell2:___________________________

Neightborhood _______________________________

Address____________________________

| **A1**. Gender | Male____, Female____, Other _____  Which one?: _________________ | | | | | | | | |
| --- | --- | --- | --- | --- | --- | --- | --- | --- | --- |
| **A2.** Age | _______ years | | | | | |  | | |
| **A3.** Date of birth | Day | | Month | | Year | | | | |
|  |  |  |  |  |  |  | |  |  |
| **A4**. Marital Status | _____ Single _____ Married  _____ Divorced  _____ Separated  _____ Widow/Widower  _____ Free Union | | | | | | | | |
| **A5**. Social security | _____ Private  _____ Public  _____ None  _____ Special regime  _____ Other _________ Especify (**A5a**_Other)  **A5b.** (Health insurer) ____________ | | | | | | | | |

| **A6.** Education level *(Select the highest education level completed)* | _____ None  _____ Primary School  _____ Secondary School  _____ Technical level  _____ University  _____ Postgraduate  Other? (**A6a**) _________ |
| --- | --- |
| **A7**. What is your work status at the moment? | _____ Full time job  _____ Part time job  _____ Independent work  _____ Study and Job  _____ Study, but do not work  _____ Neither study nor work |
| **A8.** Do you practice any physical activity? | ____ Yes (Continue to A8a - A8c)  ____ No (Go to A9) |
| **A8a.** What kind of physical activity do you practice? (Include any sport or physical therapy) | _____________________________ |
| **A8b.** How many times a week do you practice physical activity? | _____ time(s) per week |
| **A8c.** How long time do you spend in physical activity per day? | _____ minutes per day |
| **A9.** Do you use any psychoactive substance? | ___Yes (Continue to A9a) ____No (Go to A10) |
| **A9a.** What kind of psychoactive substance?  *You can select more than one option* | _____ Tobacco/Cigarette  _____ Cannabis  _____ Cocaine  _____Other  Specify (A9a_Otro) ____________ |
| **A10**. Have you been diagnosed with a disease (i.e: Diabetes, hypertension, cancer, etc.)? | ____No (Go to A11)  ____Yes Continue to A10a)  Specify (A10a_Yes) ___________ |
| **A10a.** Do you take any medicine for this disease? | ____No  ____Yes  Specify (A10a_Yes) ___________  ______________________________ |

| **A11. Neurogenic Bladder** | | |
| --- | --- | --- |
| Do you use urinary catheter? | | ____Yes  ____No (Go to A12) |
| What kind of urinary catheter do you use? | | ___ Intermittent  ___ Permanent (Go to A12) |
| How many bladder catheterizations do you do a day? | | ____ (number) |
| How often do you replace the permanent bladder catheter? | | ____ Days |
| Has or has had involuntary loss of urine due to overdistension of the bladder? | | ___ Yes  ___ No |
| **A11. *INTERVIEWER****, Does the patient have neurogenic bladder?* | | ____Yes  ____No |
| **A12. Neurogenic Bowel Dysfunction (NBD)** | | |
| Frequency of defecation? | ___ Daily (0 Points)  ___ 2-6 times every week (1 Point)  ___ Less than once a week (6 Points) | |
| Time used for each defecation? | ___ Less than 30 min. (0 Points)  ___ 31-60 min. (3 Points)  ___ More than one hour (7 Points) | |
| Uneasiness, headache or perspiration during defecation? | ___ Yes (2 Points)  ___ No (0 Points) | |
| Regular use of tablets against constipation? | ___ Yes (2 Points)  ___ No (0 Points) | |
| Regular use of drops against constipation? | ___ Yes (2 Points)  ___ No (0 Points) | |
| Digital stimulation or evacuation of the anorectum? | ___ Less than once a week (0 Points)  ___ Once or more every week (6 Points) | |
| Frequency of fecal incontinence? | ___ Daily (13 Points)  ___ 1-6 times every week (7 Points)  ___ 1-4 times every month (6 Points)  ___ Less than once every month (0 points) | |
| Medication against fecal incontinence? | ___ Yes (4 Points)  ___ No (0 Points) | |
| Flatus incontinence? | ___ Yes (2 Points)  ___ No (0 Points) | |
| Perianal skin problems? | ___ Yes (3 Points)  ___ No (0 Points) | |
| Total score (range 0-47)  **TOTAL Score** __________ | ____ Very minor (0 – 6 Points)  ____ Minor (7 – 9 Points)  ____ Moderate (10 -13 Points)  ____ Severe (14 or more) | |
| **A12.** **INTERVIEWER:** Does the patient have neurogenic bowel dysfunction? | __Yes ____No | |

| **A13**. Occurrence’s date of spinal cord injury | Day | | Month | | Year | | | |
| --- | --- | --- | --- | --- | --- | --- | --- | --- |
|  |  |  |  |  |  |  |  |  |
| **A13.a** Years since injury | _______ years | | | | | | | |
| **A14.** ASIA Impairment Scale | _____ A _____ B _____ C _____ D | | | | | | | |
| **A15.** Sensory neurological level of injury | _______ | | | | | | | |
| **A16.** Motor neurological level of injury | _______ | | | | | | | |
| **A17.** Neurological level of injury | _______ | | | | | | | |
| **A18.** Skin at pressure sites | Without ulcer ______ Active ulcer ______ | | | | | | | |
| **A19.** Currently, are you receiving pain medication? | ____Yes (Continue to A19a y A20)  ____No (go to Section B) | | | | | | | |
| **A19a**. Name of the medication? | 1. __________________ 2. __________________ 3. __________________ | | | | | | | |
| **A19b.** Current doses per day? | 1. _____ mg/day. 2. _____ mg/day. 3. _____ mg/day. | | | | | | | |
| **A19c.** When was the last time your dose was changed? | _____ More than one month ago.  _____ Less than one month ago | | | | | | | |

| **A20. Measure of Medication Adherence - Moriski Green**  (Patient is considered ADHERENT if the participant answer NO to all 4 questions) | | |
| --- | --- | --- |
|  | Yes | No |
| ¿Do you ever forget to take your medicine? |  |  |
| ¿Are you careless at times about taking you medicine? |  |  |
| When you feel better ¿Do you sometimes stop taking your medicine? |  |  |
| Sometimes if you feel worse when you take the medicine, ¿Do you stop taking it? |  |  |
| **A 20.** ¿Can be the patient classified as ADHERENT? |  |  |

1. **SECTION B: PROBABILITY OF NEUROPATHIC PAIN- DN-4 QUESTIONNAIRE**

| *The following questions are about the pain cause by the spinal cord injury.* | | | |
| --- | --- | --- | --- |
| Questions | Characteristics | Options | |
|  |  | Yes | No |
| **B1.** Does the pain have one or more of the following characteristics? | Burning |  |  |
|  | Painful Cold |  |  |
|  | Electric Shocks |  |  |
| **B2.** Is the pain associated with one or more of the following symptoms in the same area? | Tingling |  |  |
|  | Pins and Needles |  |  |
|  | Numbness |  |  |
|  | Itching |  |  |
| **B3.** Is the pain located in an area where the physical examination may reveal one or more of the following characteristics? | Hypoesthesia to touch |  |  |
|  | Hypoesthesia to pinprick |  |  |
| **B4.** In the painful area, can the pain be caused or increased by: | Brushing |  |  |

| ***B4.a. International Spinal Cord Injury Pain Classification***  *(Check mark √)* | | |
| --- | --- | --- |
| At level spinal cord injury pain | Within the three dermatomes below the level of injury | *Check mark √* |
| Below level spinal cord injury pain | More than three dermatomes below of the level of injury | *Check mark √* |

| ***B4.b. Numeric Pain Rating Scale***  *(Check mark √)*  *Please indicate the numeric value on the segmented scale that best describes their pain intensity* | | | | | | | | | | |
| --- | --- | --- | --- | --- | --- | --- | --- | --- | --- | --- |
| None | Mild | | | Moderate | | | | Severe | | |
| 0 | 1 | 2 | 3 | 4 | 5 | 6 | 7 | 8 | 9 | 10 |

1. **SECTION C: THE PAIN CATASTROPHIZING SCALE - PCS**

| *We are interested in the types of thoughts and feelings that you have when you are in pain. Listed below are thirteen statements describing different thoughts and feeling that may be associated with pain. Using the following scale, please indicate the degree to which you have these thoughts and feelings when you are experiencing pain regarding spinal cord injury.* |
| --- |

| **Painful Situations** | **Not at all** | **To a slight degree** | **To a moderate degree** | **To a great degree** | **All the time** |
| --- | --- | --- | --- | --- | --- |
| C1. I worry all the time about whether the pain will end | 0 | 1 | 2 | 3 | 4 |
| C2.I feel I can´t go on. | 0 | 1 | 2 | 3 | 4 |
| C3. It’s terrible and it’s never going to get any better | 0 | 1 | 2 | 3 | 4 |
| C4. It´s awful and I feel that it overwhelms me | 0 | 1 | 2 | 3 | 4 |
| C5. I feel I can´t stand it anymore | 0 | 1 | 2 | 3 | 4 |
| C6. I become afraid that the pain will get worse | 0 | 1 | 2 | 3 | 4 |
| C7. I keep thinking of other painful events | 0 | 1 | 2 | 3 | 4 |
| C8. I anxiously want the pain to go away | 0 | 1 | 2 | 3 | 4 |
| C9. I can´t seem to keep it out of mind | 0 | 1 | 2 | 3 | 4 |
| C10. I keep thinking about how much it hurts | 0 | 1 | 2 | 3 | 4 |
| C11. I keep thinking about how badly I want the pain to stop | 0 | 1 | 2 | 3 | 4 |
| C12.There´s nothing I can do to reduce the intensity of the pain | 0 | 1 | 2 | 3 | 4 |
| C13. I wonder whether something serious may happen | 0 | 1 | 2 | 3 | 4 |

1. **SECTION D: 12-item WORLD HEALTH ORGANIZATION DISABILITY ASSESMENT SCHEDULE (WHO DAS 2.0)**

| *In the past 30 days, how much difficulty did you have in:* |
| --- |

| **Activities** | None | Mild | Moderate | Severe | Extreme / Cannot Do |
| --- | --- | --- | --- | --- | --- |
| D1. Standing for long periods such as 30 minutes. | 1 | 2 | 3 | 4 | 5 |
| D2.Taking care of your household responsibilities | 1 | 2 | 3 | 4 | 5 |
| D3. Learning a new task, for example, how to get to a new place. | 1 | 2 | 3 | 4 | 5 |
| D4. How much of a problem did you have in joining in community activities (for example, festivities, religious or other activities) in the same way as anyone else can | 1 | 2 | 3 | 4 | 5 |
| D5. How much have you been emotionally affected by your health problems | 1 | 2 | 3 | 4 | 5 |
| D6. Concentrating on doing something for ten minutes | 1 | 2 | 3 | 4 | 5 |
| D7. Walking a long distance such as a kilometer (or equivalent) | 1 | 2 | 3 | 4 | 5 |
| D8. Washing you whole body | 1 | 2 | 3 | 4 | 5 |
| D9. Getting dressed | 1 | 2 | 3 | 4 | 5 |
| D10. Dealing with people you do not know | 1 | 2 | 3 | 4 | 5 |
| D11. Maintaining a friendship | 1 | 2 | 3 | 4 | 5 |
| D12. Your day-to-day work/school | 1 | 2 | 3 | 4 | 5 |

1. **SECTION E: THE SHORT FORM (36) HEALTH SURVEY (SF-36)**

| **SF-36** | |  |
| --- | --- | --- |
|  |  |  |
| **PLEASE ANSWER THE 36 QUESTIONS OF THE HEALTH SURVEY COMPLETELY, HONESTLY, AND WITHOUT INTERRUPTIONS** | |  |
| 1. In general, would you say your health is: | |  |
|  | 1. Excellent |  |
|  | 2. Very Good |  |
|  | 3. Good |  |
|  | 4. Fair |  |
|  | 5. Poor |  |
| 2. ¿Compared to one year, how would you rate your health in general now? | |  |
|  | 1. Much better now than one year ago |  |
|  | 2. Somewhat better now than one year ago |  |
|  | 3. About the same |  |
|  | 4.Somewhat worse now than one year ago |  |
|  | 5. Much worse than one year ago |  |
| **THIS FOLLOWING ITEMS ARE ABOUT ACTIVITIES YOU MIGHT DO DURING A TYPICAL DAY. DOES YOUR HEALTH NOW LIMIT YOU IN THESE ACTIVITIES? IF SO, HOW MUCH?** | |  |
| 3. Vigorous activities, such as running, lifting heavy objects, participating in strenuous sports. | |  |
|  | 1. Yes, limited a lot |  |
|  | 2. Yes, limited a little |  |
|  | 3. No, Not limited at all |  |
| 4. Moderate activities, such as moving a table, pushing a vacuum cleaner, bowling, or playing golf? | |  |
|  | 1. Yes, limited a lot |  |
|  | 2. Yes, limited a little |  |
|  | 3. No, Not limited at all |  |
| 5. Lifting or carrying groceries? | |  |
|  | 1. Yes, limited a lot |  |
|  | 2. Yes, limited a little |  |
|  | 3. No, Not limited at all |  |
| 6. Climbing several flights of stairs? | |  |
|  | 1. Yes, limited a lot |  |
|  | 2. Yes, limited a little |  |
|  | 3. No, Not limited at all |  |
| 7. Climbing one flight of stairs? | |  |
|  | 1. Yes, limited a lot |  |
|  | 2. Yes, limited a Little |  |
|  | 3. No, Not limited at all |  |
| 8. Bending, kneeling or stooping? | |  |
|  | 1. Yes, limited a lot |  |
|  | 2. Yes, limited a Little |  |
|  | 3. No, Not limited at all |  |
| 9. Walking more than a mile? | |  |
|  | 1. Yes, limited a lot |  |
|  | 2. Yes, limited a Little |  |
|  | 3. No, Not limited at all |  |
| 10. Walking several blocks? | |  |
|  | 1. Yes, limited a lot |  |
|  | 2. Yes, limited a little |  |
|  | 3. No, Not limited at all |  |
| 11. Walking one block? | |  |
|  | 1. Yes, limited a lot |  |
|  | 2. Yes, limited a Little |  |
|  | 3. No, Not limited at all |  |
| 12. Bathing or dressing yourself? | |  |
|  | 1. Yes, limited a lot |  |
|  | 2. Yes, limited a Little |  |
|  | 3. No, Not limited at all |  |
| **DURING THE PAST 4 WEEKS, HAVE YOU HAD ANY OF THE FOLLOWING PROBLEMS WITH YOUR WORK OR OTHER REGULAR DAILY ACTIVITIES AS A RESULT OF YOUR PHYSICAL HEALTH?** | |  |
| 13. Cut down the amount of time you spent on work or other activities? | |  |
|  | 1. Yes |  |
|  | 2. No |  |
| 14. Accomplished less than you would like? | |  |
|  | 1. Yes |  |
|  | 2. No |  |
| 15. Were limited in the kind of work or other activities? | |  |
|  | 1. Yes |  |
|  | 2. No |  |
| 16. Had difficulty performing the work or other activities (for example, it took extra effort)? | |  |
|  | 1. Yes |  |
|  | 2. No |  |
| 17. Cut down the amount of time you spent on work or other activities? | |  |
|  | 1. Yes |  |
|  | 2. No |  |
| 18. Accomplished less than you would like? | |  |
|  | 1. Yes |  |
|  | 2. No |  |
| 19. Didn't do work or other activities as carefully as usual? | |  |
|  | 1. Yes |  |
|  | 2. No |  |
| 20. Emotional problems interfered with your normal social activities with family, friends, neighbors, or groups? | |  |
|  | 1. Not at all |  |
|  | 2. Slightly |  |
|  | 3. Moderately |  |
|  | 4. Severe |  |
|  | 5. Very Severe |  |
| 21. How much bodily pain have you had during the past 4 weeks? | |  |
|  | 1. Not at all |  |
|  | 2. Slightly |  |
|  | 3. Moderately |  |
|  | 4. Severe |  |
|  | 5. Very Severe |  |
| 22. During the past 4 weeks, how much did pain interfere with your normal work (including both work outside the home and housework)? | |  |
|  | 1. Not at all |  |
|  | 2. Slightly |  |
|  | 3. Moderately |  |
|  | 4. Severe |  |
|  | 5. Very Severe |  |
| **THESE QUESTIONS ARE ABOUT HOW YOU FEEL AND HOW THINGS HAVE BEEN WITH YOU DURING THE LAST 4 WEEKS. FOR EACH QUESTION, PLEASE GIVE THE ANSWER THAT COMES CLOSEST TO THE WAY YOU HAVE BEEN FEELING** | |  |
| 23. Did you feel full of pep? | |  |
|  | 1. All of the time |  |
|  | 2. Most of time |  |
|  | 3. A Good bit of the time |  |
|  | 4. Some of the time |  |
|  | 5. A little bit of the time |  |
|  | 6. None of the time |  |
| 24. Have you been a very nervous person? | |  |
|  | 1. All of the time |  |
|  | 2. Most of time |  |
|  | 3. A Good bit of the time |  |
|  | 4. Some of the time |  |
|  | 5. A little bit of the time |  |
|  | 6. None of the time |  |
| 25. Have you felt so down in the dumps that nothing could cheer you up?? | |  |
|  | 1. All of the time |  |
|  | 2. Most of time |  |
|  | 3. A Good bit of the time |  |
|  | 4. Some of the time |  |
|  | 5. A little bit of the time |  |
|  | 6. None of the time |  |
| 26. Have you felt calm and peaceful? | |  |
|  | 1. All of the time |  |
|  | 2. Most of time |  |
|  | 3. A Good bit of the time |  |
|  | 4. Some of the time |  |
|  | 5. A little bit of the time |  |
|  | 6. None of the time |  |
| 27. Did you have a lot of energy? | |  |
|  | 1. All of the time |  |
|  | 2. Most of time |  |
|  | 3. A Good bit of the time |  |
|  | 4. Some of the time |  |
|  | 5. A little bit of the time |  |
|  | 6. None of the time |  |
| 28. Have you felt downhearted and blue? | |  |
|  | 1. All of the time |  |
|  | 2. Most of time |  |
|  | 3. A Good bit of the time |  |
|  | 4. Some of the time |  |
|  | 5. A little bit of the time |  |
|  | 6. None of the time |  |
| 29. Did you feel worn out? | |  |
|  | 1. All of the time |  |
|  | 2. Most of time |  |
|  | 3. A Good bit of the time |  |
|  | 4. Some of the time |  |
|  | 5. A little bit of the time |  |
|  | 6. None of the time |  |
| 30. Have you been a happy person? | |  |
|  | 1. All of the time |  |
|  | 2. Most of time |  |
|  | 3. A Good bit of the time |  |
|  | 4. Some of the time |  |
|  | 5. A little bit of the time |  |
|  | 6. None of the time |  |
| 31. Did you feel tired? | |  |
|  | 1. All of the time |  |
|  | 2. Most of time |  |
|  | 3. A Good bit of the time |  |
|  | 4. Some of the time |  |
|  | 5. A little bit of the time |  |
|  | 6. None of the time |  |
| 32. During the past 4 weeks, how much of the time has your physical health or emotional problems interfered with your social activities (like visiting with friends, relatives, etc.)? | |  |
|  | 1. All of the time |  |
|  | 2. Most of time |  |
|  | 3. A Good bit of the time |  |
|  | 4. Some of the time |  |
|  | 5. A little bit of the time |  |
|  | 6. None of the time |  |
| **HOW TRUE OR FALSE IS EACH OF THE FOLLOWING STATEMENTS FOR YOU?** | |  |
| 33. I seem to get sick a little easier than other people. | |  |
|  | 1. Definitely true |  |
|  | 2. Mostly true |  |
|  | 3. Don´t know |  |
|  | 4. Mostly false |  |
|  | 5. Definitely false |  |
| 34. I am as healthy as anybody I know. | |  |
|  | 1. Definitely true |  |
|  | 2. Mostly true |  |
|  | 3. Don´t know |  |
|  | 4. Mostly false |  |
|  | 5. Definitely false |  |
| 35. I expect my health to get worse | |  |
|  | 1. Definitely true |  |
|  | 2. Mostly true |  |
|  | 3. Don´t know |  |
|  | 4. Mostly false |  |
|  | 5. Definitely false |  |
| 36. My health is excellent | |  |
|  | 1. Definitely true |  |
|  | 2. Mostly true |  |
|  | 3. Don´t know |  |
|  | 4. Mostly false |  |
|  | 5. Definitely false |  |

**NOTE TO THE INTERVIEWER: FILL UP ONLY IF IT IS THE SECOND MEASUREMENT**

1. **COMMENTS ABOUT THE INTERVIEW**

| Please briefly indicate, if you had any kind of difficult to attending the therapies during your care process: |
| --- |
|  |

| Please briefly, write your opinion on the therapies during your care process. |
| --- |
|  |

1. **END OF QUESTIONNAIRE.**

***THANK YOU FOR YOUR VALUABLE PARTICIPATION!***

Ending Time: ________________
