# Supplementary material for: Effectiveness of Hydrotherapy on Neuropathic Pain and Pain Catastrophization in Patients With Spinal Cord Injury: Protocol for a Pilot Trial Study
Source: JMIR Res Protoc. 2022 Apr 29;11(4):e37255. doi: 10.2196/37255 (PMC9107053; doi:10.2196/37255)
Supplement: Multimedia Appendix 5 [file resprot_v11i4e37255_app5.docx]

**Appendix 5. Checklist of inclusion and exclusion criteria**

Patient's name: _____________________

Date of verification:

| Day | | Month | | Year | | | |
| --- | --- | --- | --- | --- | --- | --- | --- |
|  |  |  |  |  |  |  |  |

This list will be verified at the INITIAL assessment in the first medical consultation.

Check / Ask these questions to the patient:

| Questions | Yes | Not |
| --- | --- | --- |
| 1. Are you under 18 years old? |  |  |
| 1. Do you have pressure sores at this time? |  |  |
| 1. Have you had a fever in the last 48 hours? |  |  |
| 1. Do you have a gastrostomy, tracheostomy, or permanent urinary catheter? |  |  |

Check the following:

| Item | Yes | Not |
| --- | --- | --- |
| 1. Is the patient classified as ASIA E? |  |  |
| 1. Does the patient have cognitive impairment or deficit? |  |  |

In case of **YES** as answer to any of the questions and items, there was at least one exclusion criteria and therefore the patient is not eligible to participate in the study.

Patients answering **YES** to the presence of fever (Question C), will be directed to receive medical attention at their primary care practice or provider.
