# Supplementary material for: Effectiveness of Hydrotherapy on Neuropathic Pain and Pain Catastrophization in Patients With Spinal Cord Injury: Protocol for a Pilot Trial Study
Source: JMIR Res Protoc. 2022 Apr 29;11(4):e37255. doi: 10.2196/37255 (PMC9107053; doi:10.2196/37255)
Supplement: Multimedia Appendix 6 [file resprot_v11i4e37255_app6.docx]

**Appendix 6. Randomization protocol**

In a simple random assignment, small sample size may generate unbalance between Intervention and Control groups. Thus, assignment in balanced blocks (length fixed, four cells per block) will be performed.

Since it is expected to include approximately 48 patients and each block has four cells, we will use 12 blocks, as follows:

Blocks = Number of patients/Number of cells per block

Blocks = 48/4 = 12

As there are only two treatment alternatives (Physical Therapy [**PF**] and hydrotherapy [**H**]), each one of the 12 blocks, include one of six possible combinations:

| **H** |  | **PT** |  | **H** |  | **PT** |  | **H** |  | **PT** |
| --- | --- | --- | --- | --- | --- | --- | --- | --- | --- | --- |
| **H** |  | **PT** |  | **PT** |  | **H** |  | **PT** |  | **H** |
| **PT** |  | **H** |  | **H** |  | **PT** |  | **PT** |  | **H** |
| **PT** |  | **H** |  | **PT** |  | **H** |  | **H** |  | **PT** |

The order in which they would be used to generate the random sequence was assigned by simple randomization, as the following example:

**Figure 1. Figure 2.**

| **Random number table** | | | |  | **4** |  | **1** |  | **3** |  | **2** |  | **6** |  | **5** |
| --- | --- | --- | --- | --- | --- | --- | --- | --- | --- | --- | --- | --- | --- | --- | --- |
| 4119531 | 4988078 | 1219531 | 4988078 |  | **H** |  | **PT** |  | **H** |  | **PT** |  | **H** |  | **PT** |
| 5632359 | 6333025 | 5632359 | 3233025 |  | **H** |  | **PT** |  | **PT** |  | **H** |  | **PT** |  | **H** |
| 8735761 | 3215335 | 8735761 | 5815335 |  | **PT** |  | **H** |  | **H** |  | **PT** |  | **PT** |  | **H** |
| 9606428 | 5343794 | 4106428 | 7843794 |  | **PT** |  | **H** |  | **PT** |  | **H** |  | **H** |  | **PT** |
| 5377195 | 2840052 | 7020053 | 9167023 |  |  |  |  |  |  |  |  |  |  |  |  |
| 3247026 | 5967257 | 1682752 | 8688276 |  |  |  |  |  |  |  |  |  |  | | |
| 1031519 | 9757768 | 9745772 | 6437154 | 1. | **PT** | 5. | **PT** | 10. | **H** | 14. | **H** | 18. | **PT** | 22. | **H** |
| 4385344 | 9517134 | 2116949 | 4459841 | 2. | **PT** | 6. | **H** | 11. | **PT** | 15. | **H** | 19. | **H** | 23. | **PT** |
| 3185277 | 2756192 | 2122874 | 9488321 | 3. | **H** | 7. | **PT** | 12. | **H** | 16. | **PT** | 20. | **H** | 24. | **PT** |
| 6399024 | 8509009 | 7020053 | 2476303 | 4. | **H** | 8. | **H** | 13. | **PT** | 17. | **PT** | 21. | **PT** | 25. | **H** |

A table of random numbers was generated in Excel, 16 columns by 30 rows, with numbers made up of 6 digits. A starting point was randomly selected within the table, and it was defined to advance two columns to the right and three rows upwards for the selection of the number (See ***Figure 1***)

Since the balanced blocks were 12, once it stopped on the number, the first digit (of that number) or the first two digits were selected (one digit from 1 to 9, or two digits from 10 to 12). In the example, the first selected block will go in position 4, second block will go in position 1, then moves in the stipulated direction to the next number, and continue with third block in the 3^rd^ position and the fourth block on the position 2. Once the order of all 12 blocks has been assigned, the sequence is organized and the treatments will be assigned (See ***Figure 2***).
