# Supplementary material for: Effectiveness of Hydrotherapy on Neuropathic Pain and Pain Catastrophization in Patients With Spinal Cord Injury: Protocol for a Pilot Trial Study
Source: JMIR Res Protoc. 2022 Apr 29;11(4):e37255. doi: 10.2196/37255 (PMC9107053; doi:10.2196/37255)
Supplement: Multimedia Appendix 7 [file resprot_v11i4e37255_app7.docx]

**Appendix 7. Standard physical therapy protocol**

Management of neuropathic pain (NP) in people with spinal cord injury (SCI), in addition to pharmacological management, includes various therapeutic modalities which include: massage, osteopathy, acupuncture, exercise, transcranial electrical stimulation, transcutaneous electrical nerve stimulation (TENS), hypnosis, cognitive therapy, and transcranial magnetic stimulation, among others [1].

The protocol described below will be used in the control group of a study that seeks to compare the effect of hydrotherapy on neuropathic pain with the therapy that is conventionally used in our setting, physical therapy.

Control group will carry out a total of 18 standard physical therapy sessions for the management of NP, distributed in 2 weekly sessions, for a period of 9 weeks, each session lasting 45 minutes.

Each physical therapy session will include as recommended and commonly used, 10 minutes of stretching, 15 minutes of aerobic exercise, and 20 minutes of strengthening exercises [2,3]

The application of TENS will not be included in the protocol for the control group, as it is not considered a standard therapy and because it requires daily application, up to 3 times per day, for a minimum of two weeks [4,5]

The intervention for the control group will be carried out by the physiotherapists who usually see patients with SCI in the institution, under the supervision of a physiotherapist specialized in neurorehabilitation, a member of the group of researchers, who, prior the intervention, carried out an 8 hours training with physiotherapists, in order to unify intervention criteria.

The physiotherapists in charge of care will actively participate in the construction of the protocol, defining the exercises to be performed according to the spaces and equipment available in the unit. In addition, in order to guarantee that during the completion of each physical therapy session, all participants in the study undergo all the scheduled interventions, a checklist containing all the interventions performed will be completed at the end of each session.

As the population to be treated will be made up of people with SCI with neurological levels from C4 who have already completed their rehabilitation process, training in functional activities will not be the goal and the sessions will be carried out in groups, with a relationship of 2 patients by physical therapist during each session.

All participants will perform self-stretching exercises of the 4 limbs and the trunk, aerobic exercises with upper extremities, and strengthening exercises for the upper extremities and trunk, according to the neurological level.

**Basic considerations for structuring the exercise protocol:**

Below, some basic considerations while structuring the intervention are listed, taking advantage of the best available evidence, having Lisa Harvey as a reference, who in her book “Treatment of spinal cord injury. Guide for physiotherapists”, makes a complete and updated review of the aspects deal with in chapters 8, 9, and 12.

**1. AEROBIC EXERCISE:**

Good cardiovascular fitness is important to maintain a good general condition and a good quality of life in the long term [6,7-9]

To grade the **intensity** of the exercise, the Borg effort scale [10,11-13] was selected, which is based on the patient's perception of effort, working with intensities between levels 12 and 16. It was selected to push the wheelchair itself alternated with repetitive transfer exercises [14,16] in cycles of 3 minutes separated by short periods of rest.

The training will be done in a group way to improve the motivation of the patients

**2. MUSCLE STRENGTHENING EXERCISES:**

Muscle strength training within this protocol will be aimed at the muscles that keep their innervation intact according to the previously established neurological level.

There is still much debate about optimal protocols for progressive resistance training [16,17-23]. For this protocol, considering that the patient with SCI, in addition to strength, needs resistance and speed to carry out daily functional activities, the following training scheme was established:

- The muscle groups to be strengthened will work in movement patterns where they exercise in a similar way to how they should work in daily activities, combining concentric, eccentric, and isometric contractions and adjusting the speed to make it similar to that of functional activities [24,25].

- 3 series of 8 to 12 repetitions will be performed, adjusting the resistance to work at approximately 60-80% of the maximum resistance [24,26,27]

- Recovery periods of 30-second between series.

- Participants will work with their own body weight with the help of elastic bands when required, varying the loads to make training more efficient [28,29]

- During all training sessions, exercises will be included for the shoulder and scapula muscles, the latissimus dorsi, the brachial triceps, and the trunk muscles that preserve their innervation.

**3.STREAMS:**

Contractures, or lack of joint mobility, are common complications of SCI and may be due to neurologic compromise, prolonged rest, and soft tissue changes.

Due to the short intervention times and the need to include within a session: aerobic and strength training, and the stretching of various muscle groups to guarantee the maintenance of optimal ranges of joint mobility, within this protocol, all the selected stretches will be carried out, performing 3 self-stretches sustained 10 seconds for each muscle group, in order to teach the patient how to do it three times a week at home, performing 10 repetitions for each muscle group.

Selected muscles: latissimus dorsi, abdominals and spinals, glutes, hamstrings, hip adductors, hip rotators, iliopsoas, and plantiflexors

**REFERENCES:**

1. Mehta S, Teasell RW, Loh E, Short C, Wolfe DL, Benton B HJ. Pain Management - Spinal Cord Injury Research Evidence. 2016; 1–92. Available from: https://scireproject.com/evidence/rehabilitation-evidence/pain-management/
2. Ginis KAM, Latimer AE, McKechnie K, Ditor DS, Hicks AL, Bugaresti J. Using exercise to enhance subjective well-being among people with spinal cord injury: The mediating influences of stress and pain. Rehab Psychol 2003; 48: 157-64.
3. Ditor DS, Latimer AE, Ginis KA, Arbor KP, McCartney N, Hicks AL. Maintenance of exercise participation in individuals with spinal cord injury: effects on quality of life, stress and pain. Spinal Cord 2003; 41: 446-50.
4. Özkul Ç, Kilinç M, Yildirim SA, Topçuo ʇ lu EY, Akyüz M. Effects of visual illusion and transcutaneous electrical nerve stimulation on neuropathic pain in patients with spinal cord injury: A randomized controlled cross-over trial. J Back Musculoskelet Rehabil. 2015; 28 (4): 709–19

1. Norrbrink C. Transcutaneous electrical nerve stimulation for treatment of spinal cord injury neuropathic pain. J Rehab Res Dev 2009; 46: 85-93.

1. Noreau L, Shephard RJ: Spinal cord injury, exercise and quality of life. Sports Med 1995; 20: 226–250

1. Duran FS, Lugo L, Ramirez L et al: Effects of an exercise program on the rehabilitation of patients with spinal cord injury. Arch Phys Med Rehabil 2001; 82: 1349–1354.

1. Davis G, Glaser RM: Cardiorespiratory fitness following spinal cord injury. In Ada L, Canning C (eds): Key Issues in Neurological Physiotherapy. Oxford, Butterworth Heinemann, 1990: 155–196.

1. Martin Ginis KA, Hicks AL: Exercise research issues in the spinal cord injured population. Exerc Sport Sci Rev 2005; 33: 49–53.

1. Stewart MW, Melton-Rogers SL, Morrison S et al: The measurement properties of fitness measures and health status for persons with spinal cord injuries. Arch Phys Med Rehabil 2000; 81: 394–400.

1. Borg G: Psychophysical basis of perceived exertion. Med Sci Sports Exerc 1982; 14: 371–381.

1. Borg G: Borg's Perceived Exertion and Pain Scales. Champaign, IL, Human Kinetics, 1998.

1. Capodaglio P, Grilli C, Bazzini G: Tolerable exercise intensity in the early rehabilitation of paraplegic patients. A preliminary study. Spinal Cord 1996; 34: 684–690

1. Franklin BA: Exercise testing, training and arm ergometry. Sports Med 1985; 2: 100-119.

1. Tordi N, Dugue B, Klupzinski D et al: Interval training program on a wheelchair ergometer for paraplegic subjects. Spinal Cord 2001; 39: 532–537.

1. Campos GE, Luecke TJ, Wendeln HK et al: Muscular adaptations in response to three different resistance-training regimens: specificity of repetition maximum training zones. Eur J Appl Physiol 2002; 88: 50–60.

1. Munn J, Herbert RD, Hancock MJ et al: Resistance training for strength: effect of number of sets and contraction speed. Med Sci Sports Exerc 2005; 37: 1622–1626.

1. Feigenbaum MS, Pollock ML: Prescription of resistance training for health and disease. Med Sci Sports Exerc 1999; 31: 38–45.

1. Rhea MR, Alvar BA, Burkett LN et al: A meta-analysis to determine the dose response for strength development. Med Sci Sports Exerc 2003; 35: 456–464.

1. Rhea MR, Alvar BA, Burkett LN: Single versus multiple sets for strength: a meta-analysis to address the controversy. Res Q Exerc Sport 2002; 73: 485–488.

1. Carpinelli RN: Berger in retrospect: effect of varied weight training programs on strength. Br J Sports Med 2002; 36: 319–324.

1. Pereira MI, Gomes PS: Movement velocity in resistance training. Sports Med 2003; 33: 427–438.

1. Carpinelli RN, Otto RM: Strength training single versus multiple sets. Sports Med 1998; 26: 73–84.

1. Kraemer WJ, Ratamess NA: Fundamentals of resistance training: progression and exercise prescription. Med Sci Sports Exerc 2004; 36: 674–688.

1. Sale D, MacDougall D: Specificity in strength training: a review for the coach and athlete. Can J Appl Sport Sci 1981; 6: 87–92.

1. Rhea MR, Alvar BA, Burkett LN et al: A meta-analysis to determine the dose response for strength development. Med Sci Sports Exerc 2003; 35: 456–464.

1. Hoeger WW, Barette SL, Hale DF et al: Relationship between repetitions and selected percentages of one repetition maximum. J Appl Sport Sci Res 1987; 1: 11–13.

1. Fleck SJ: Periodized strength training: a critical review. J Strength Cond Res 1999; 13: 82–89.

1. Kraemer WJ: A series of studies - the physiological basis for strength training in American football: fact over philosophy. J Strength Cond Res 1997; 11: 131–142.
