# Supplementary material for: Effectiveness of Hydrotherapy on Neuropathic Pain and Pain Catastrophization in Patients With Spinal Cord Injury: Protocol for a Pilot Trial Study
Source: JMIR Res Protoc. 2022 Apr 29;11(4):e37255. doi: 10.2196/37255 (PMC9107053; doi:10.2196/37255)
Supplement: Multimedia Appendix 8 [file resprot_v11i4e37255_app8.docx]

**Appendix 8. Hydrotherapy protocol**

**Initial considerations:**

- Patients with neurogenic bladder, should perform bladder catheterization before and after admission to the pool.
- Patients with fecal incontinence should wear plastic disposable diaper to prevent leakage of fecal matter and contaminate the pool.
- Assistance from the physiotherapist, assistant, and familiar for immersion in the pool, depending on the patient functionality and general condition.
- Availability of flotation devices for each patient, if the patient requires it.
- Adaptation of the patient to the aquatic environment in the first two sessions, reducing the risk of phobia of water and perception of insecurity.

Pool conditions:

- Water temperature between 31 °C and 34 °C.
- Maximum depth of the pool of 1.40 meters.

Length of intervention in water:

- 60 minutes

**Adaptation of the patient to the aquatic environment**

Familiarization activities are carried out in the water, such as:

- Immersion holding their breath for 30 seconds
- Changing from Prone to Supine assisted by physiotherapist
- Transitions floating horizontally to vertically and if possible, adoption of bipedal posture
- Making bubbles blowing without swallowing water
- Displacements for 20 to 30 seconds with assistance and without assistance if possible, keeping the head out of the water. These displacements may also be using the wall or pool ladder, or in the middle of the pool.

**Phase I. Initial phase**

Length: 10 minutes

*Breath control and relaxation exercises*

- Lip-sealing activities: when going underwater, release bubble through the nose
- Diaphragmatic breathing with the assistance of verbal commands and tactile stimulation from the physiotherapist
- Buoyancy activities
- Watsu’s Technique

This will be done based on Halliwick’s method.

**Phase II. Central stage**

Length: 40 minutes

*Balance and postural control - 20 minutes*

- Supine and upright, do exercises of sagittal, transverse, and longitudinal rotation
- Supine and upright, do combined rotation exercises with sagittal and longitudinal axis, and transverse with longitudinal axis
- Postural changes from supine to prone, prone to supine, and from supine and prone to vertical
- Exercises that involve activities of static control and central postural control
- Exercises of central postural control with turbulence and tactile stimuli by the physiotherapist
- Exercises of motion with movement facilitated by the physiotherapist
- Exercises in supine, with symmetrical and asymmetrical movements of upper limbs
- Exercises in prone, with symmetrical and asymmetrical movements of upper limbs
- Exercises for muscles of the neck, shoulder girdle, upper limbs, trunk, pelvic girdle, and lower limbs (depending on the level of injury and neurological involvement) using isometric muscle activities, isotonic and isokinetic with the Bad Ragaz’s Technique
- Activities of static and dynamic balance with horizontal and vertical floating
- Activities with eyes open and closed

*Aerobic Exercise - 20 minutes*

- Moderate intensity exercises according to the perception of effort based on the Borg scale (6-20) from 11 (soft) to 13 (somewhat stronger)
- Exercises of swimming patterns depending on the skill of each patient with Halliwick’s technique
- Exercises involving immersion, anterior, side, and rear displacements, based on the conditions and capacities of each individual patient
- Bicycle exercises in water with support in the upper limbs, mobilizing the trunk, pelvis or lower limbs depending on the level of injury and neurological compromise
- Intervallic exercises in circuit, with activities involving the muscles of neck, shoulder girdle, upper limbs, trunk, pelvic girdle, and lower limbs, depending on the level of injury and neurological involvement
- Exercises using resistance devices and flotation

** The progression of activities and exercises depends on the conditions of each patient.*

**Phase IIl final**

Length: 10 minutes

- Relaxation exercises - Muscle stretches with Watsu’s technique for upper limb muscles, shoulder girdle, neck, pelvic girdle, lower limbs, paraspinal, and abdominal muscles
- Breathing exercises. Based on Halliwick’s technique.

**References:**

- American College of Sports Medicine (Ed. 9 ). (201 4 ). *Guidelines for exercise testing and prescription*. Williams & Wilkins.

- Hospital Universitario de Evaristo García ESE Valle Practical Guide in aquatic therapy. Code: GI-MFR-035. 31/01 / 2018. Version: 000. Available Intranet HUV - Daruma .

- Coppieters , MW, & Nee, RJ (2012). Neurodynamics : Movement for neuropathic pain states. In *Fascia: The Tensional Network of the Human Body*(pp 425-432.). Churchill Livingstone.

- Frye, SK, Ogonowska-Slodownik , A., & Geigle, PR (2017). Aquatic Exercise for People With Spinal Cord Injury. *Archives of physical medicine and rehabilitation*, *98*(1), 195-19

- Gorman, PH, Scott, W., VanHiel , L., Tansey, KE, Sweatman , WM, & Geigle, PR (2019). Comparison of peak oxygen consumption response to aquatic and robotic therapy in chronic motor Individuals with incomplete spinal cord injury: a randomized controlled trial. *Spinal cord*1.

- Güeita , J., Alonso, M., & Fernandez , C. (2015). *Aquatic therapy. Collisions from physiotherapy and occupational therapy*. Elsevier Publishing House.

- Lambeck , J. (2017). Hydrotherapy in adult neurology. *Medical EWAC http: // www. ewac . com. Accessed*, *10*.

- Moscoso Alvarado, F. (2006). Aquatic therapy in neurorehabilitation . *Rev. Colomb . rehabil*, *1*(5), 101-111.

- Zivi , I., Maffia , S., Ferrari, V., Zarucchi , A., Molatore , K., Maestri , R., & Frazzitta , G. (2018). Effectiveness of aquatic versus land physiotherapy in the treatment of peripheral neuropathies: a randomized controlled trial. *Clinical rehabilitation*, *32*(5), 663-670.
