# Supplementary material for: Effectiveness of Hydrotherapy on Neuropathic Pain and Pain Catastrophization in Patients With Spinal Cord Injury: Protocol for a Pilot Trial Study
Source: JMIR Res Protoc. 2022 Apr 29;11(4):e37255. doi: 10.2196/37255 (PMC9107053; doi:10.2196/37255)
Supplement: Multimedia Appendix 9 [file resprot_v11i4e37255_app9.docx]

**Appendix 9. Adverse event report form**

Patient ID: _____________________

Assigned Group: Physical Therapy _______

                            Hydrotherapy ________

1. Date of occurrence of the event:

| DD | | MM | | YYYY | | | |
| --- | --- | --- | --- | --- | --- | --- | --- |
|  |  |  |  |  |  |  |  |

Time: _______________

Place of occurrence: __________________________________________

1. Event reporting date

| DD | | MM | | YYYY | | | |
| --- | --- | --- | --- | --- | --- | --- | --- |
|  |  |  |  |  |  |  |  |

Time: _______________

Name of the person reporting the event: ________________________________________

Title of the person reporting the event: __________________________________________

1. Event to report (Mark with an X)

Faintness: ____ Fall: _____ Drowning:____ Dermatitis: _____Pneumonia:_____ Lipothymia: ____

Other?:__________________________________________________________________________

1. Event description:

|  |
| --- |
|  |
|  |
|  |
|  |
|  |
|  |
|  |

1. Procedure to attend the event:

|  |
| --- |
|  |
|  |
|  |
|  |
|  |
|  |

1. Event analysis:

Responsible for the analysis: __________________________________________________________

Classification of the event (**See Table 1 - Below**):_________________________________________

1. Unsafe actions:

|  |
| --- |
|  |
|  |
|  |
|  |
|  |
|  |

1. Corrective measures:

|  |
| --- |
|  |
|  |
|  |
|  |
|  |
|  |
|  |

| **Table 1.**Types of event. | |
| --- | --- |
| Adverse event | It is the result of a health care intervention that unintentionally caused harm. |
| Preventable adverse event | Unwanted and unintended result that would have been avoided by complying with the care standards available at a given time. |
| Non-preventable adverse event | Unwanted and unintended result that occurs despite compliance with the care standards. |
| Incident | It is an event or circumstance that happens in clinical care and does not cause harm to the patients, but in its occurrence incorporate failures into the care processes. |
| Complication | It is the damage or unexpected clinical result not attributable to healthcare but to the disease or the patient's own conditions. |

***Project details:***

- Name: Effectiveness of hydrotherapy on neuropathic pain and pain catastrophization in patients with spinal cord injury:

- Type of study: Randomized controlled clinical trial

- Institutions: Departamento de Medicina Física y Rehabilitación. Grupo Cátedra de Discapacidad y Rehabilitación. Grupo de investigación Sinergia. Fundación SCISCO.

.
