# Supplementary material for: Effectiveness of Hydrotherapy on Neuropathic Pain and Pain Catastrophization in Patients With Spinal Cord Injury: Protocol for a Pilot Trial Study
Source: JMIR Res Protoc. 2022 Apr 29;11(4):e37255. doi: 10.2196/37255 (PMC9107053; doi:10.2196/37255)
Supplement: Multimedia Appendix 10 [file resprot_v11i4e37255_app10.docx]

**Appendix 10. Form: cause for withdrawal**

Patient ID: _____________________

Assigned Group: Physical Therapy _______

                            Hydrotherapy _______

Date of report :

| Day | | Month | | Year | | | |
| --- | --- | --- | --- | --- | --- | --- | --- |
|  |  |  |  |  |  |  |  |

Mark the failure to follow-up with an **X.**

| **Definition** | Yes |
| --- | --- |
| **Voluntary Withdrawal:** People included in the trial who did not want or could not continue in the study. |  |
| **Withdrawal by indication:** Patient who meets exclusion criteria or requires in-hospital management of any pathology. |  |
| **Loss to follow-up:** Patient included in the trial who did not attend the indicated therapy and from whom it was not possible to obtain follow-up data. That is, the participants with whom after the 4 week of intervention it was not possible to communicate to perform the follow-up assessment. |  |
| **Protocol deviation**: Patients who, due to different circumstances, did not follow the assigned protocol when changing intervention groups. |  |

**Description of the case:**

________________________________________________________________________________________________________________________________________________________________________________________________________________________________________________________________________________________________________________________________

Responsible for making the report: ______________________________________

| Position | Mark with an X |
| --- | --- |
| Therapist |  |
| Investigator |  |
| Other |  |
