# Supplementary material for: Effectiveness of Hydrotherapy on Neuropathic Pain and Pain Catastrophization in Patients With Spinal Cord Injury: Protocol for a Pilot Trial Study
Source: JMIR Res Protoc. 2022 Apr 29;11(4):e37255. doi: 10.2196/37255 (PMC9107053; doi:10.2196/37255)
Supplement: Multimedia Appendix 11 [file resprot_v11i4e37255_app11.docx]

**Appendix 11. Informed consent**

[*translated from Spanish*]

**Neuropathic Pain in Patients with Spinal Cord Injury, Cali - Colombia**

You are invited to participate in a study carried out by the Department of Physical Medicine and Rehabilitation at Universidad del Valle and the Hospital Universitario del Valle (HUV). We are trying to identify the effectivity of physical therapy and hydrotherapy to treat neuropathic pain, a pain that is not caused by a physical reason but something in your nerves’ connections, in approximately 40 people with spinal cord injury. We would like to invite you to participate and we want to know if you would be interested in participating in our study.

To know the effects of the therapies we have to follow the steps that I am about to describe, which will take approximately three months:

**Study steps:**

*First step.* If you decide to participate, you will answer a survey run by an interviewer from our team. The survey will allow us to know demographic data, clinical situations related with your disease such as treatment, complications, sexual and reproductive health, quality of life, and pain. This survey is voluntary and no identifiable information will be collected on it. Instead, we will use an alpha-numeric code to identify your information. Then, you will be evaluated by a physician specialized in physical medicine and rehabilitation to assess sensitivity and muscle strength.

If you are eligible to participate, you will be randomly assigned to one of the two following groups:

- A group receiving initially aquatic therapy (hydrotherapy)

- or a group receiving initially physical therapy

Each group will receive at the end of the first type of therapy the other therapy so everyone will have access to both type of interventions.

*Second step:* After the survey and random assignation to a group, you will receive two weekly sessions of therapies (according to the group you have been assigned) during 9 weeks for a total of 18 sessions. All therapies in both groups will be provided by professionals in physical rehabilitation at the Hospital Universitario del Valle. During your treatment you may receive phone calls from our team to ask about your perception of pain and health status as well as to plan your next visit to the hospital.

*Third step:* In addition to the phone calls, when you finish the treatment, you will be cited to the hospital to answer the survey and being medically evaluated again, as in the first step. We will schedule a medical appointment for you in which you will be evaluated again by the physiatrist (physician specialized in physical medicine and rehabilitation).

It is important for you to know that some people may feel uncomfortable answering some survey questions. If you chose to participate in this study, you are not required to answer the questions that make you feel uncomfortable, you are not required to receive the intervention during the 9 weeks, and you can choose to leave the study at any time. Your responsibility is to answer in the most honest way and abide by the indications of the professionals during the therapy sessions for your safety, as well as the rules for the use of the facilities.

Hydrotherapy is a physical therapy modality that is used in patients with spinal cord injury. Neither hydrotherapy, nor conventional physical therapy implies any risk for the people who receives or performs it. You will benefit in receiving personalized attention and orientation to enhance your clinical condition.

It is also important that you know, that you will not receive any financial benefit. Our team will give you a transportation allowance to help you guarantee your attendance to the 18 therapy sessions and citations to answer surveys and physical examination. This transportation allowance will be of $12.000 Colombia pesos (COP, approximately 3 US dollars [USD]) per visit up to a total of $216.000 COP (approximately $54 USD). It will be provided once you finish each session.

All identifiable information that you provide to our research group will be changed with codes instead of using identification numbers or names. The data collected is only for research purposes and its handling will be exclusive by the researchers. Additionally, you will be given a copy of this informed consent document to keep in case of concerns.

In case that there is any change in the steps of the study previously mentioned, you will be informed immediately.

If you have any questions or concerns regarding this study, please do not hesitate to contact us. For more information, please contact Maria Ana Tovar Sanchez, Chief of the Department of Physical Medicine and Rehabilitation, Universidad del Valle at +57 312 7842335 (attn. Andres Reyes) or with Sara Gabriela Pacichana-Quinayáz at +57 312 212 8016, the study coordinator. The address of the study office in Cali is Carrera 5ta # 36 - 08, Department of Physical Medicine and Rehabilitation, First floor. Hospital Universitario del Valle. You can also contact the staff of the Institutional Review Board of the Universidad del Valle by phone: at +57 2 518-5677 ext. 4077 or to the e-mail: [eticasalud@correounivalle.edu.co](mailto:eticasalud@correounivalle.edu.co).

In order for you to participate in our study, you must sign this document. If you prefer not to sign, you can mark with an "X" in the signature part of this document, which indicates that you freely agree to participate in the study. In the same way, we need the signatures of two people to tell us that you decide to freely participate in the study.

_____________________________

Participant (ID):

Witness 1 _________________

Witness 2 _________________

Finally, we request your authorization so that this collected information can be used in other similar research studies in the future, with the prior approval of the Ethics Committee of the Universidad del Valle.

I accept YES_____ or NO_______
