# Supplementary material for: Effectiveness of Hydrotherapy on Neuropathic Pain and Pain Catastrophization in Patients With Spinal Cord Injury: Protocol for a Pilot Trial Study
Source: JMIR Res Protoc. 2022 Apr 29;11(4):e37255. doi: 10.2196/37255 (PMC9107053; doi:10.2196/37255)
Supplement: Multimedia Appendix 12 [file resprot_v11i4e37255_app12.pdf]

UNIVERSIDAD DEL VALLE
Vicerrectoría de Investigaciones

FORMATO DE EVALUACIÓN DE PROPUESTAS DE INVESTIGACIÓN

Datos generales del Proyecto:

Título del proyecto:
EFECTIVIDAD DE LA HIDROTERAPIA PARA DISMINUCIÓN DE INTENSIDAD Y CATASTROFIZACIÓN DE DOLOR NEUROPÁTICO EN PACIENTES CON TRAUMA RAQUIMEDULAR

Investigador Principal:

MARIA ANA TOVAR SANCHEZ ,

Coinvestigadores:
GLORIA PATRICIA ARANGO HOYOS , SARA GABRIELA PACICHANA QUINAYáZ ,

Facultad o Instituto académico:
SALUD

Unidad Académica:
MEDICINA FISICA Y REHABILITACION ,

1. Justificación de la propuesta

1. 1 Justificación y pertinencia

¿Se argumenta la naturaleza y magnitud del problema de tal forma que es clara la importancia científica y tecnológica de la investigación en la producción del conocimiento? ¿Es relevante, adecuada y oportuna en términos de su contribución al desarrollo de la región y del país y/o a la consolidación de la comunidad científica o artística?

Es una propuesta pertinente con importancia en el ámbito local

2. Concordancia y calidad de los planteamientos

2. 1 Pregunta o problema de investigación

¿La pregunta o problema de investigación está formulado de manera adecuada y precisa?

La pregunta se formula de manera adecuada

2. 2 Objetivo general y objetivos específicos

¿Los objetivos son claros y coherentes con el planteamiento del problema o pregunta de investigación?

Los objetivos son claros, sin embargo el primer objetivo es una actividad que siempre debe realizarse en una investigación y que por lo tanto algunos consideran no es necesario incluirla, por que no ayuda a responder la pregunta que se esta planteando

2. 3 Metodología

¿La propuesta plantea una metodología clara, coherente y factible con el problema y presenta en forma organizada y precisa, cómo se alcanzará cada uno de los objetivos propuestos?

La metodología es clara y pertinente

2. 4 Marco teórico y estado del arte

¿Se presenta un marco teórico bien construido acompañado de una síntesis del contexto general (nacional y/o mundial) en el cual se ubica el tema de la propuesta: estado actual, vacios a llenar, etc.?

se identifican vacíos de conocimiento

2. 5 Concordancia

¿Existe concordancia entre la pregunta o problema de investigación, los objetivos y la metodología propuesta?

si existe concordancia

3. Productos

3. 1 Productos esperados

¿Los resultados describen los productos teóricos o prácticos, bienes o servicios que se pueden lograr con la realización del proyecto?

Los productos esperados son realizables

3. 2 Estrategias de divulgación

¿Explicita el compromiso de publicación científica y de divulgación a través de estrategias concretas?

Se especifican los productos esperados y estos son acordes con el desarrollo del proyecto

4. Impactos

4. 1 Se evidencian impactos que se generen con el proyecto a corto, mediano y largo plazo?

Se evidencian impactos que se generen con el proyecto a corto, mediano y largo plazo?

Se describen de manera adecuada los impactos del proyecto y los plazos en los que se espera se presenten

TABLA DE CALIFICACIÓN DE CRITERIOS ESPECIFICOS PARA EVALUACIÓN DE PROYECTOS DE INVESTIGACIÓN

Escala de calificación: 1 a 5, utilizando dos decimales (5=máxima calificación)

Criterio	Nota por criterio	Ponderación (100%)	Nota ponderada
Impacto	4.50	10%	0.45
Justificación y pertinencia	4.45	15%	0.67
Marco teórico y aporte al estado del arte	4.45	15%	0.67
Métodos	4.50	20%	0.90
Objetivos	4.20	20%	0.84
Pregunta o problema de investigación	4.60	20%	0.92
**Total**		**100%**	**4.45**

(1) Los valores definidos en la columna de ponderación fueron establecidos por el Comité Central de Investigaciones por lo tanto no son susceptibles de modificación en la evaluación.

LOS SIGUIENTES APARTES DEBERÁN SER EVALUADOS SOLO EN CASO DE QUE SE REQUIERA MEDIANTE LA CARTA DE SOLICITUD DE EVALUACIÓN.

1. Cronograma

¿La secuencia de actividades y tiempo previsto para su realización son adecuados para alcanzar los resultados esperados?

La secuencia de actividades esta descrita de forma adecuada

2. Presupuesto

Existe concordancia entre el presupuesto total, las actividades, los objetivos y resultados planteados del proyecto? ¿Muestra justificación adecuada de los rubros, cantidades y montos solicitados con los objetivos, la metodología y la duración del proyecto?

No aplica

3. Permisos, licencias o contratos

¿El proyecto requiere permisos, licencias o contratos en el marco de la normativa ambiental nacional vigente relacionada con los proyectos de investigación en áreas de las ciencias biológicas, agrarias, ambientales y biomédicas?

No

CALIFICACIÓN DE OTROS CRITERIOS PARA EVALUACIÓN DE PROYECTOS DE INVESTIGACIÓN

Escala de calificación: 1 a 5, utilizando dos decimales (5=máxima calificación)

Cronograma	4.5
Presupuesto	5
Permisos, licencias o contratos	5

Recomendación final

Es un proyecto adecuadamente formulado, con potencial de generar impacto en el área del conocimiento

APRUEBA________ NO APRUEBA________ REQUIERE MODIFICACIONES________

Evaluador No. 1
Nombre del evaluador **Firma**

Fecha: (DD/MM/AA): 10/09/2018
