# Supplementary material for: Effectiveness of Hydrotherapy on Neuropathic Pain and Pain Catastrophization in Patients With Spinal Cord Injury: Protocol for a Pilot Trial Study
Source: JMIR Res Protoc. 2022 Apr 29;11(4):e37255. doi: 10.2196/37255 (PMC9107053; doi:10.2196/37255)
Supplement: Multimedia Appendix 13 [file resprot_v11i4e37255_app13.pdf]

UNIVERSIDAD DEL VALLE
Vicerrectoría de Investigaciones

FORMATO DE EVALUACIÓN DE PROPUESTAS DE INVESTIGACIÓN

Datos generales del Proyecto:

Título del proyecto:
EFECTIVIDAD DE LA HIDROTERAPIA PARA DISMINUCIÓN DE INTENSIDAD Y CATASTROFIZACIÓN DE DOLOR NEUROPÁTICO EN PACIENTES CON TRAUMA RAQUIMEDULAR

Investigador Principal:

MARIA ANA TOVAR SANCHEZ ,

Coinvestigadores:
GLORIA PATRICIA ARANGO HOYOS , SARA GABRIELA PACICHANA QUINAYáZ ,

Facultad o Instituto académico:
SALUD

Unidad Académica:
MEDICINA FISICA Y REHABILITACION ,

1. Justificación de la propuesta

1. 1 Justificación y pertinencia

¿Se argumenta la naturaleza y magnitud del problema de tal forma que es clara la importancia científica y tecnológica de la investigación en la producción del conocimiento? ¿Es relevante, adecuada y oportuna en términos de su contribución al desarrollo de la región y del país y/o a la consolidación de la comunidad científica o artística?

El proyecto presenta una descripción del problema coherente, que permite establecer la importancia científica del mismo. Además, expone la búsqueda de soluciones que redunden en el bienestar del paciente.

2. Concordancia y calidad de los planteamientos

2. 1 Pregunta o problema de investigación

¿La pregunta o problema de investigación está formulado de manera adecuada y precisa?

Si bien el proyecto no presenta una pregunta de investigación propiamente dicha, el planteamiento que se hace en este apartado deja ver lo que el investigador propone desarrollar

2. 2 Objetivo general y objetivos específicos

¿Los objetivos son claros y coherentes con el planteamiento del problema o pregunta de investigación?

Los objetivos planteados están conexos con el planteamiento del problema. Además estos están en concordancia con las hipótesis que se plantean.

2. 3 Metodología

¿La propuesta plantea una metodología clara, coherente y factible con el problema y presenta en forma organizada y precisa, cómo se alcanzará cada uno de los objetivos propuestos?

La metodología planteada es muy clara y organizada. Plantea las actividades a desarrollar de manera meticulosa (tipo de estudio, calculo de la muestra y desarrollo de actividades) y con los soportes requeridos para ello. Hay que precisar un aspecto menor en los criterios de inclusión con relación a la población. En principio establecen que la población son adultos entre los 21-49 años y en los criterios de inclusión hablan de adultos a partir de los 18 años. Modificar si es posible.

2. 4 Marco teórico y estado del arte

¿Se presenta un marco teórico bien construido acompañado de una síntesis del contexto general (nacional y/o mundial) en el cual se ubica el tema de la propuesta: estado actual, vacios a llenar, etc.?

En este apartado del documento, el marco teórico muestra una concreta y congruente redacción en cuanto a las definiciones interpretadas en los diferentes ítems. Sin embargo, no se aprecia el estado del arte de este estudio (que se ha hecho, aplicación de otras técnicas, en otros tipos de lesión) Todo esto hace que el trabajo tome mucho más relevancia, pues le daría la originalidad que este pueda tener.

2. 5 Concordancia

¿Existe concordancia entre la pregunta o problema de investigación, los objetivos y la metodología propuesta?

Existe concordancia entre los apartados descripción del problema, objetivos y metodología

3. Productos

3. 1 Productos esperados

¿Los resultados describen los productos teóricos o prácticos, bienes o servicios que se pueden lograr con la realización del proyecto?

El proyecto plantea una serie de productos como resultados del desarrollo del mismo. Sin embargo como sugerencia menor se solicita revisar si el ítem de publicaciones se puede cumplir en la meta de un año tal y como se plantea en el documento

3. 2 Estrategias de divulgación

¿Explicita el compromiso de publicación científica y de divulgación a través de estrategias concretas?

El proyecto propone el sometimiento de manuscritos para su publicación (dos artículos Q3) y la presentación en un evento científico como estrategia de divulgación

4. Impactos

4. 1 Se evidencian impactos que se generen con el proyecto a corto, mediano y largo plazo?

Se evidencian impactos que se generen con el proyecto a corto, mediano y largo plazo?

Si se evidencian impactos que se generan con el proyecto. Sin embargo, en algunos casos es complejo que se puedan dar en ese tiempo (ej., publicación de artículos).

TABLA DE CALIFICACIÓN DE CRITERIOS ESPECIFICOS PARA EVALUACIÓN DE PROYECTOS DE INVESTIGACIÓN

Escala de calificación: 1 a 5, utilizando dos decimales (5=máxima calificación)

Criterio	Nota por criterio	Ponderación (100%)	Nota ponderada
Impacto	4.00	10%	0.40
Justificación y pertinencia	4.00	15%	0.60
Marco teórico y aporte al estado del arte	3.00	15%	0.45
Métodos	4.00	20%	0.80
Objetivos	5.00	20%	1.00
Pregunta o problema de investigación	4.00	20%	0.80
**Total**		**100%**	**4.05**

(1) Los valores definidos en la columna de ponderación fueron establecidos por el Comité Central de Investigaciones por lo tanto no son susceptibles de modificación en la evaluación.

LOS SIGUIENTES APARTES DEBERÁN SER EVALUADOS SOLO EN CASO DE QUE SE REQUIERA MEDIANTE LA CARTA DE SOLICITUD DE EVALUACIÓN.

1. Cronograma

¿La secuencia de actividades y tiempo previsto para su realización son adecuados para alcanzar los resultados esperados?

Las actividades del cronogramas son adecuadas y alcanzables

2. Presupuesto

Existe concordancia entre el presupuesto total, las actividades, los objetivos y resultados planteados del proyecto? ¿Muestra justificación adecuada de los rubros, cantidades y montos solicitados con los objetivos, la metodología y la duración del proyecto?

No se recibió formato de presupuesto del proyecto

3. Permisos, licencias o contratos

¿El proyecto requiere permisos, licencias o contratos en el marco de la normativa ambiental nacional vigente relacionada con los proyectos de investigación en áreas de las ciencias biológicas, agrarias, ambientales y biomédicas?

El proyecto no requiere de permisos ambientales

CALIFICACIÓN DE OTROS CRITERIOS PARA EVALUACIÓN DE PROYECTOS DE INVESTIGACIÓN

Escala de calificación: 1 a 5, utilizando dos decimales (5=máxima calificación)

Cronograma	5
Presupuesto	0
Permisos, licencias o contratos	5

Recomendación final

De ser posible tener en cuenta modificar aspectos menores del proyecto como la edad en los criterios de inclusión (teniendo en cuenta la población) y el impacto a corto plazo respecto a publicaciones

APRUEBA________ NO APRUEBA________ REQUIERE MODIFICACIONES________

Evaluador No. 2
Nombre del evaluador **Firma**

Fecha: (DD/MM/AA): 10/09/2018
